# Suppressing Phase Segregation and Improving Stability in Mixed-Halide Perovskites through Spinel Oxide-Directed Epitaxy

**DOI:** 10.1021/jacs.6c04846

**Published:** 2026-06-15

**Authors:** Diana K. LaFollette, Martin Gomez-Dominguez, Kunal Datta, Amanda Conde del Moral, Seungjun Cha, Jack Lawton, Ruipeng Li, Benjamin Lawrie, Yanqi Luo, Carlo A. R. Perini, Guoxiang Hu, Juan-Pablo Correa-Baena

**Affiliations:** † School of Materials Science and Engineering, 1372 Georgia Institute of Technology, Atlanta, Georgia 30332, United States; ‡ National Synchrotron Light Source II, 8099 Brookhaven National Laboratory, Upton, New York 11973, United States; § Center for Nanophase Materials Science, 6146 Oak Ridge National Laboratory, Oak Ridge, Tennessee 37831, United States; ∥ Materials Science and Technology Division, Oak Ridge National Laboratory, Oak Ridge, Tennessee 37831, United States; ⊥ Advanced Photon Source, 530313 Argonne National Laboratory, Lemont, Illinois 60439, United States; # School of Chemistry and Biochemistry, Georgia Institute of Technology, Atlanta, Georgia 30332, United States

## Abstract

Mixed-cation mixed-halide perovskite compositions are essential for achieving the required bandgaps for high-efficiency multijunction photovoltaics, yet their stability remains limited by interfacial defects, phase segregation, and degradation. Here, we introduce spinel oxides as a new family of lattice-matched substrates that enable crystalline, phase-pure, compositionally-uniform, bromide-rich perovskite film growth. The effect of spinel oxides is two-fold: reducing defects at the bottom interface by templating film growth and inducing beneficial compressive strain through mismatch-dependent substrate-perovskite lattice coupling. Spinel oxide substrates facilitate growth of highly crystalline films and eliminate detrimental secondary phases across thicknesses. Using grazing incidence X-ray diffraction, X-ray fluorescence, cathodoluminescence–scanning electron microscopy, cryogenic photoluminescence, and density functional theory, we reveal that Mg-halide bonds at the bottom interface induce lattice mismatch-dependent compressive strain that suppresses halide segregation and further reduces defect formation. In addition, films grown on spinel oxides maintain over 87% of the perovskite phase after 12 h under 100% relative humidity, as monitored by in situ grazing incidence wide-angle X-ray scattering (GIWAXS), compared to less than 70% for control samples. This work extends lattice matching from vapor-deposited epitaxial semiconductors to solution-processed halide perovskites to establish a broadly applicable strategy for defect suppression, phase homogenization, and long-term stability. Based on the fundamental science explored here, we set the stage for the development of lattice-matched spinel oxide charge transport layers to be integrated into perovskite solar cells and other optoelectronic devices.

## Graphical abstract

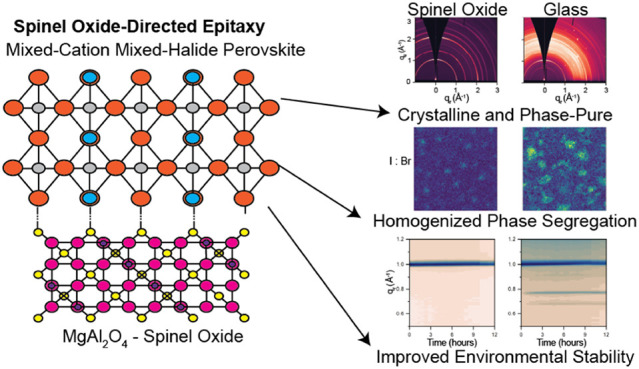

## Introduction

Lead halide perovskites (LHPs) are promising materials for many optoelectronic applications, including light emission, lasing, and photodetectors, because of their tunable band gaps, long carrier lifetimes, defect tolerance, and ease of fabrication.^1–3^ One of the most commercially promising LHP applications is in multijunction photovoltaics, where wide-bandgap perovskite solar cell(s) (PSC) are combined with narrow-bandgap solar cell(s) (silicon, CIGS, organic, or other PSC) to overcome the single-junction Shockley–Queisser limit, increasing efficiencies and specific power, and reducing the cost of solar electricity.^4–11^ However, multijunction applications require wide bandgaps only achievable with bromide-rich mixed-halide compositions.^12^ These bromide-rich compositions consistently suffer from defects that lead to compositional heterogeneity and complex phase transformations resulting in high rates of nonradiative recombination and significant open-circuit voltage (*V*_*oc*_) losses.^7,13–18^ Initially, photoinduced phase segregation was considered the main cause of *V*_oc_ losses due to the formation of low-energy trap states.^19^ However, more recent works suggest that poor crystallinity, nanoscale compositional heterogeneity, and defects play a more significant role in performance losses.^1,9,12,14,20,21^ This work aims to stabilize and homogenize bromide-rich compositions for multijunction applications by engineering the bottom interface using lattice-matched spinel oxides, a novel material family for applications in PSCs.

To achieve the required bandgap for a desired optoelectronic application, we use compositional engineering: changing the atoms at the A- and X-sites in the LHP ABX_3_ structure.^22–24^ Most state-of-the-art PSCs use some combination of A-site cations (methylammonium (MA), formamidinium (FA), and/or cesium (Cs)), B-site metal cation (lead (Pb)), and X-site halides (iodide (I) and/or bromide (Br)).^3,21^ However, our past works show that replacing even <20% I with Br changes phase transformations and thermal stability, while the compositions required for multijunction applications use up to 67% Br.^11,16,25^ To stabilize these complex systems, we must go beyond changing composition to engineer crystallization. We take inspiration from works that engineer crystallization for compositional homogeneity using precursor additives or self-assembled monolayers, leveraging structural templating to reduce defects at the bottom interface and induce compressive strain, thereby preventing undesirable chemical processes such as phase segregation and phase transformations.^9,26,27^

The phase segregation and degradation processes that plague bromide-rich LHPs are known to initiate at the interfaces due to their high densities of defect states, such as ionic vacancies and interstitials.^1,28,29^ The top interface has been the subject of extensive efforts to passivate defects and prevent degradation.^30–34^ However, the similarly defective bottom interface has been the focus of fewer studies. Trapped solvent voids at the bottom interface can cause disordered crystallization that limits stability and device performance.^35,36^ Depth-dependent compositional gradients due to halide-dependent crystallization kinetics can cause *V*_*oc*_ losses that limit device performance.^37,38^ We set out to reduce defects at the bottom interface and their associated performance losses by facilitating highly ordered, defect-free film growth through lattice matching with spinel oxides.

Lattice matching, using a layer with the desired crystal structure to template the growth of the following layers, is commonly employed in traditional semiconductors to induce heteroepitaxial growth.^39^ In PSC applications, lattice matching with a variety of materials, including 2D LHPs, alkali salts, and metal oxides, has been shown to improve structural properties and device performance.^40–43^ Pulsed laser deposition (PLD) of MAPbI_3_ on lattice-matched KCl single crystals achieved epitaxial growth with excellent optoelectronic properties.^44^ Lattice-matched electron transport layers (ETLs), such as SrTiO_3_ and SrSnO_3_, have improved structural stability, slowed carrier recombination, and reduced defect densities.^36,45^ With lattice-matched SrSnO_3_, migration of I from the LHP into the ETL was reduced, and long-term stability improved.^36^ In most cases, however, the benefits of lattice matching are restricted to improvements in structure and charge transport. In this work, we show that even partially coherent lattice matching between bromide-rich LHPs and spinel oxides can not only template structure but also prevent intrinsic phase segregation and support long-term stability by improving bottom interface quality and inducing compressive strain.

This work uses spinel oxides to stabilize degradation-prone bromide-rich LHP compositions for multijunction photovoltaic applications. Grazing incidence wide-angle X-ray scattering (GIWAXS) is used to characterize the structure of LHP films, demonstrating improved phase purity and preferential orientation when fabricated on spinel oxides compared with glass. Grazing incidence X-ray diffraction (GIXRD) demonstrates mismatch-dependent compressive strain induced by spinel oxide-LHP lattice coupling across a range of LHP compositions. Elemental and optical mapping show the effect of lattice coupling on homogenizing intrinsic phase segregation and improving optoelectronic properties in 50–120 nm thick films. Density functional theory (DFT) calculations provide evidence of the thermodynamic favorability of Mg-halide bonds between LHPs and spinel oxides that increases the barrier for defect formation and ion migration. By improving the film quality at the bottom interface and limiting ion migration, lattice coupling, in turn, improves stability under humidity. Our work is the first known to demonstrate that a bottom interface modification can reduce bulk phase segregation of bromide-rich compositions and establishes spinel oxides as a promising family of materials for applications in LHPs for PSCs and broader optoelectronic applications.

## Results and Discussion

### Spinel Oxides as Promising Candidates for Templating LHP Crystallization

For one material to template the heteroepitaxial growth of another, they must have similar lattice parameters and chemical natures.^46^ We can use the difference (δ) in their lattice parameters (a_substrate_, a_film_) as a preliminary assessment of whether one material can effectively template the growth of another (eq 1).^47^ Heteroepitaxial growth has been shown to occur with δ up to 9%.^48^ With very small δ, a perfectly coherent interface can be formed, but as δ increases, a semicoherent interface will form, with dislocations accommodating the increased strain.^46,48^

1 $$delta\left(\delta \right)=\frac{a_{substrate}-a_{film}}{a_{film}}$$

Spinel oxides are promising candidates to template LHP film growth as due to their similar and tunable lattice parameters, chemical compatability, and ability to be deposited via scalable deposition techniques like PLD.^49–51^ We chose MgAl_2_O_4_ as the first spinel oxide to lattice match LHPs due to its commercial availability as a substrate and its similar lattice parameter (8.1 Å) to the *a* and *b* lattice parameters of the CsFAPbIBr β-perovskite.^15,49^

The MgAl_2_O_4_ lattice can provide a template for crystalline film growth through Magnesium (Mg)–halide bonds, on either cation-halide (Figure 1a) or lead-halide terminated LHP surfaces (Figure 1b). The schematic shows an incomplete unit cell to clearly the visualize the alignment of cations and halides with the spinel oxide structure. LHPs fabricated on glass, which does not template crystallization, are used as the control (Figure 1c). Both spinel oxides and LHPs have tunable lattice parameters, providing a wide compositional space of potential material combinations (Figure 1d). The exact amount of offset between the LHP and MgAl_2_O_4_ depends on the composition selected. If heteroepitaxial growth occurs with a higher δ, then greater compressive strain would be induced, which has been shown to be beneficial for charge transport and structural stability.^52,53^ However, the higher the δ, the less coherent the substrate-LHP interface will be, and the more dislocations will be present.^46^ The focus of this work is to establish the fundamental chemistry-structure-property relationships of spinel oxides and LHPs to set the stage for future device integration. MgAl_2_O_4_, the selected prototypical spinel oxide, is used as a single crystal for the purposes of this work. Ultimate applications would combine this work’s mechanistic understanding with integration of a vapor-deposited lattice-matched spinel oxide layer with the proper band alignment for a charge transport layer, such as NiCo_2_O_4_ or Co_3_O_4_, into a full optoelectronic device.^51,54–57^ Depositing a spinel oxide as the hole transport layer in an inverted device architecture could impart the beneficial effects of lattice matching to the perovskite above while facilitating charge transport and extraction. The material insights developed here are invaluable in establishing the promise of such future endeavors.

**Figure 1:**
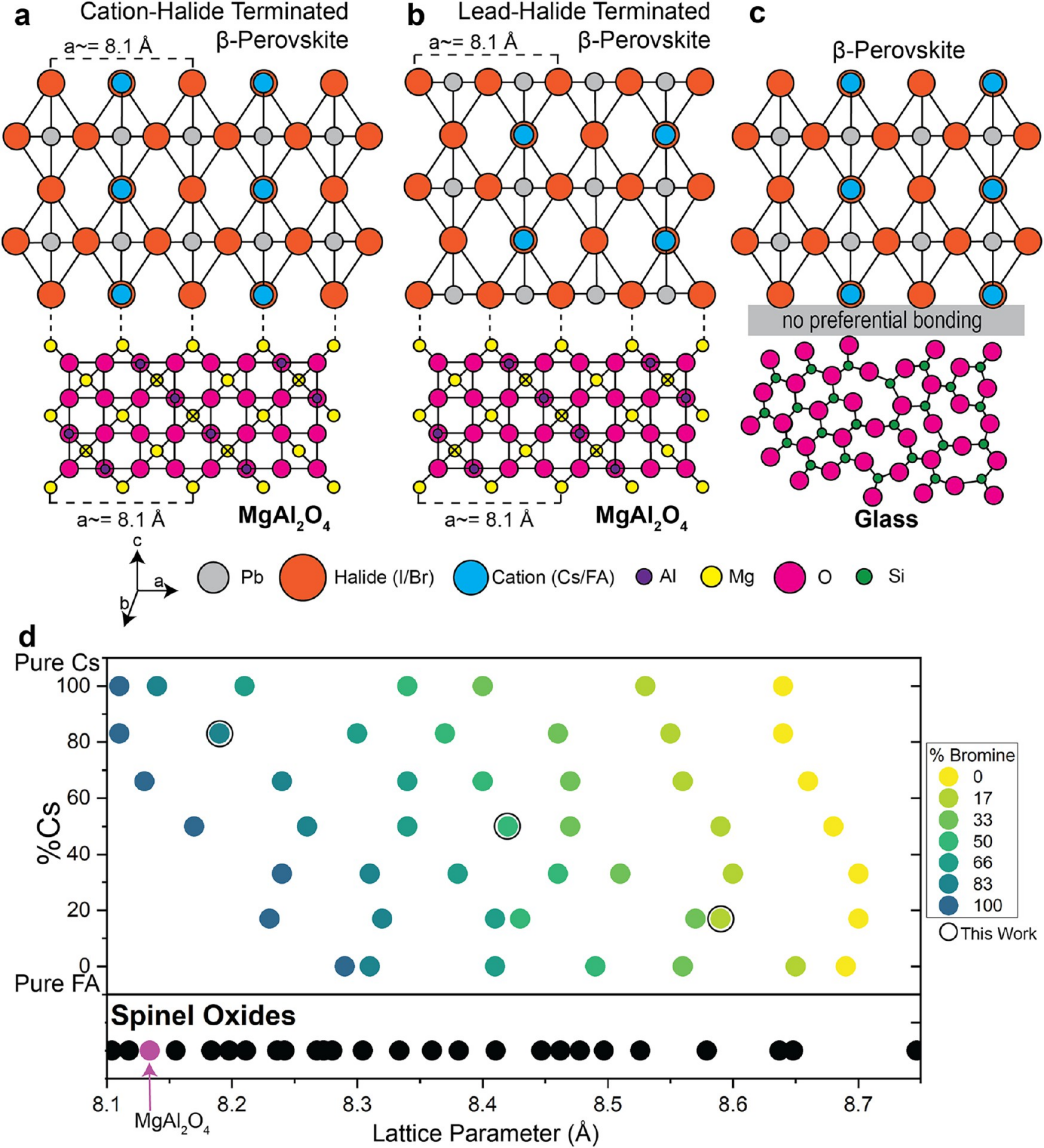
Templating LHP growth with spinel oxides. Schematic of bonds that could facilitate heteroepitaxial growth between the (110) tetragonal LHP and (100) MgAl_2_O_4_ planes on either (a) cation-halide terminated LHP surfaces or (b) lead-halide terminated LHP surfaces. (c) Schematic of LHP crystallization on glass with no preferential bonding. (d) Lattice parameters of spinel oxides and Cs-FA Pb I–Br LHPs.^15,49^ The spinel oxide used for this paper (MgAl_2_O_4_) is marked in pink, while LHP compositions are circled in black. Listed lattice parameters for spinel oxides and mixed-cation mixed-halide LHPs shown in Figure 1d are listed in Tables S1 and S2.

To study the effect of varying δ on lattice matching between spinel oxides and LHPs, we chose three different LHP compositions (black circles) shown in Figure 1d and listed in Table 1. Mixed-cation mixed-halide compositions were selected to span from popular compositions for single-junction PSCs (Cs_0.17_FA_0.83_Pb­(I_0.83_Br_0.17_)_3_ - Cs17Br17)^15^ to the smallest possible δ (Cs_0.83_FA_0.17_Pb­(I_0.17_Br_0.83_)_3_ - Cs83Br83). All δ values are within the possible range to facilitate heteroepitaxial growth^48^ and should induce in-plane compressive strain when bonded with the substrate. We fabricated LHP films by spin-coating on a spinel oxide substrate and compared them to films fabricated on glass (Figure 1a–c). LHPs in PSCs are typically fabricated on glass/transparent conducting oxide, such as fluorinated tin oxide (FTO).^3^ We first confirmed that there is no difference in crystallization between films made on amorphous glass and glass/FTO (Figure S1); thus, glass was used as the control to avoid the influence of SnO_2_ scattering peaks. It is also important to consider the smoothness of the substrate surface for lattice matching;^44^ we used polished, commercially available MgAl_2_O_4_ substrates with a roughness of less than 5 Å.^58^ Both glass and spinel oxide substrates are highly wettable, as confirmed by contact angle measurements, supporting the conclusion that changes in crystallization are due to templating rather than other chemical effects such as surface energy (Figure S2).

**Table 1:** Selected LHP Compositions and Calculated Deltas (*δ*)

Composition	delta (δ)
Cs_0.17_FA_0.83_Pb(I_0.83_Br_0.17_)_3_ – Cs_17_Br_17_	6%
Cs_0.50_FA_0.50_Pb(I_0.50_Br_0.50_)_3_ – Cs_50_Br_50_	4%
Cs_0.83_FA_0.17_Pb(I_0.17_Br_0.83_)_3_ – Cs_83_Br_83_	1.3%

### Effect of Spinel Oxides on Crystallinity, Phase Purity, and Strain

We used grazing incidence wide-angle X-ray scattering (GIWAXS) to structurally characterize LHP thin films fabricated on glass and spinel oxide (MgAl_2_O_4_) across a range of compositions and corresponding δs (Figure 2). MgAl_2_O_4_ substrates produce crystalline LHP thin films, as evidenced by the characteristic β-perovskite peaks at *q*_*r*_ = 1 Å^–1^, 1.4 Å^–1^, 2 Å^–1^.^59^ LHPs on MgAl_2_O_4_ have reduced peaks below *q*_*r*_ = 1 Å^–1^ compared to LHP films on glass, as seen in 2D images and boxed regions in 1D circular averages (Figure 2). These low-angle peaks are attributed to undesirable secondary phases PbI_2_, 4H-FAPbI_3_, and δ-CsPbI_3_ (indexed in Figure S3).^16,24,59–61^ This shows that spinel oxides suppress secondary phase formation and improve phase purity across a range of LHP compositions and δs, with reproducible results across many samples (Figures S4–S7).

**Figure 2:**
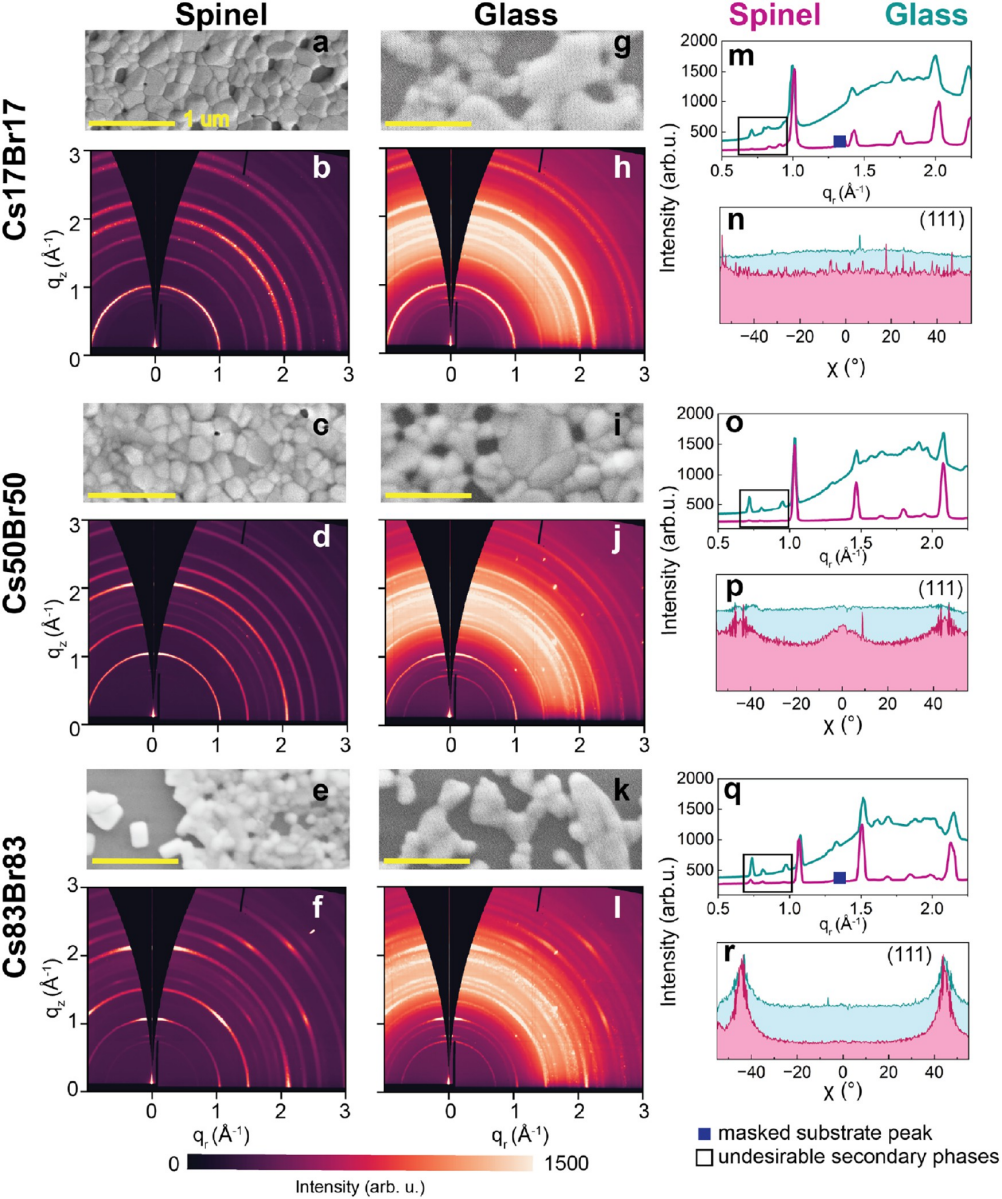
Spinel oxides improve phase purity over a range of lattice mismatches. SEM images (scale bar: 1 μm) and 2D GIWAXS patterns (α = 0.5 ^◦^) of 80 nm thin films fabricated on spinel oxides and glass for (a,b,g,h) Cs17Br17, (c,d,i,j) Cs50Br50, and (e,f,k,l) Cs83Br83 show that MgAl_2_O_4_ improves crystallinity and phase purity. Circular averages show reduced secondary phases (boxed areas) on spinel oxide (pink) compared to glass (teal) for (m) Cs17Br17, (o) Cs50Br50, and (q) Cs83Br83. The MgAl_2_O_4_ (111) peak is masked (blue square), full patterns are shown in Figure S3. Azimuthal integrations (n,p,r) on MgAl_2_O_4_ (spinel, pink) and glass (teal) for each LHP composition show changing orientation of the (111) LHP plane.

Films on glass have a high amorphous background above *q*_*r*_ = 1.4 Å^–1^ (Figure 2h,j,l), both due to the amorphous substrate itself and the discontinuous LHP film coverage compared to films on spinel oxide. Nucleation and growth processes that affect resultant morphology in heteroepitaxial growth are complex and depend on lattice mismatch as well as the relative strengths of interactions between adsorbed atoms compared to those between the atoms and the substrate.^46^ Larger δs favor island growth,^48^ but as composition and δ cannot be individually isolated, the chemical interactions between the LHP and substrate will also change, leading to complex nucleation and growth relationships that are outside the scope of this work. Processing conditions (solvent, spin-coating protocol, precursor solution concentration) are kept constant across perovskite compositions, to focus on the effect of varying δ, rather than individual process optimization. Individual process optimization would be the focus of future works with device integration.

Traditionally, heteroepitaxial growth produces highly oriented single crystals.^41^ However, solution processing, even on lattice-matched substrates, produces polycrystalline thin films due to film growth that initiates from nucleation sites, as seen in SEM images (Figure 2a,c,e,g,i,k, Figure S8).^62–64^ Even without the formation of single-crystal domains often associated with vapor deposition techniques, solution processing can facilitate bonds with the substrate to induce changes in the orientation of crystallographic domains.^41,65–67^ Orienting the 100 (cubic) or 110 (tetragonal) LHP plane perpendicular to the substrate (parallel to the direction of charge transport in a device) has shown to improve current generation and charge transport.^68–70^ To understand whether interactions between the (110) plane of the LHP and the (100) plane of the MgAl_2_O_4_ substrate change polycrystalline thin film orientation, we examine azimuthal integrations of the (111) Debye–Scherrer ring, *q*_*r*_ = 1.4 Å^–1^ (Figure 2n,p,r). A flat line, like in Figure 2n for Cs17Br17, indicates no preferential orientation of the (111), also seen as rings rather than spots in Figure 2b,h.^68,71^ Cs50Br50 is randomly oriented on glass, but has peaks at χ = −45 ^◦^, 0 ^◦^, and 45 ^◦^ on MgAl_2_O_4_ substrates (Figure 2p), demonstrating that there is preferential orientation of the (111) in two directions, both parallel and perpendicular to the substrate. These two preferential orientations are expected, as both the a and b LHP lattice parameters can match with the MgAl_2_O_4_ (100). Due to the configuration of grazing incidence measurements, the reconstruction of the Ewald sphere has a missing wedge near *q_r_* = 0, wherein there is no data available.^71,72^ To comprehensively characterize the data considering the missing wedge, azimuthal integrations should be done over two separate peaks.^68^ Azimuthal integrations of the Cs50Br50 (110) show primary orientation perpendicular to the substrate with small peaks at χ = ±45^◦^ (Figure S9), supporting changes in orientation due to the spinel oxide substrate. Cs83Br83 (Figure 2r) has peaks around χ = ±45 ^◦^ that are sharper for films on MgAl_2_O_4_, indicating orientation of the (111) perpendicular to the substrate due to lattice coupling.

As MgAl_2_O_4_ has a slightly smaller lattice parameter than the β-perovskite, successful lattice matching would induce slight in-plane compressive strain. The degree of induced compressive strain should correspond to the δ between the LHP composition and the substrate, with the highest δ (Cs17Br17) inducing the most compressive strain. In LHPs, compressive strain has been experimentally demonstrated to improve charge carrier transport, reduce ion migration, and improve structural stability.^52,62,73,74^ To assess residual strain across LHP compositions and δs, we used grazing incidence X-ray diffraction (GIXRD). The *sin* ^2^ *ψ* method is commonly used to measure residual strain in polycrystalline thin films, including LHPs, by examining the shift in a diffraction peak as a function of sample tilt to probe changes in in-plane and out-of-plane strain.^64,74,75^ Detailed methods are shown in Supplemental Note 1. Figure 3a–c shows diffraction of the 110 family of planes for Cs17Br17, Cs50Br50, and Cs83Br83. For all compositions, as ψ increases (probing planes with a larger in-plane component), the peak shifts to lower 2θ values. This shift indicates a decrease in *d*-spacing consistent with in-plane compressive strain. Peak positions are plotted as a function of *sin* ^2^ *ψ* and fitted with a linear regression model to quantify in-plane strain. In-plane registry is used to evaluate lattice matching; bonding with the spinel oxide substrate should constrain the *a* and *b* lattice parameters. All compositions have negative slopes, indicating in-plane compressive strain, and the magnitude of the slope increases with lattice mismatch (δ) (Table 1). Cs17Br17 has the highest degree of induced compressive strain (δ = 6%, slope = −0.0894), followed by Cs50Br50 (δ = 4%, slope = −0.0528), and then Cs83Br83 (δ = 1.3%, slope = −0.0356). To maximize angular sensitivity to shifts due to strain, the highest order diffraction peak giving a significant signal was used. The (220) for Cs17Br17 had reduced crystallinity and thus could not be measured; the (110) is provided instead. Because the (110) diffraction peak is at a lower 2θ, the strain-induced shift is less sensitive. Regardless, Cs17Br17 has the highest slope of the three compositions, suggesting greater induced compressive strain corresponding to lattice coupling with the highest degree of lattice mismatch. Comparisons of *sin* ^2^ *ψ* measurements on glass are shown in Figure S10. Plots of slope (approximate strain magnitude) versus δ are shown in Figure S11, supporting mismatch-dependent substrate-induced strain. Films grown on the MgAl_2_O_4_ spinel oxide substrates have significantly higher slopes than films on glass, which exhibit much smaller residual strains that are likely due to thermal annealing. The increase in compressive strain with increasing δ supports the hypothesis of substrate-induced lattice coupling. However, fully coherent heteroepitaxial growth would require all 3 compositions to converge to the same in-plane lattice parameter when ψ = 90 ^◦^, which is not observed. This indicates partial strain relaxation, likely accommodated by misfit dislocations, as expected for heteroepitaxial growth above the critical thickness.^76,77^ We will refer to this effect as lattice coupling, to reflect the mismatch-dependent, substrate-induced strain from LHP/spinel oxide interactions that results in a semicoherent interface.

**Figure 3:**
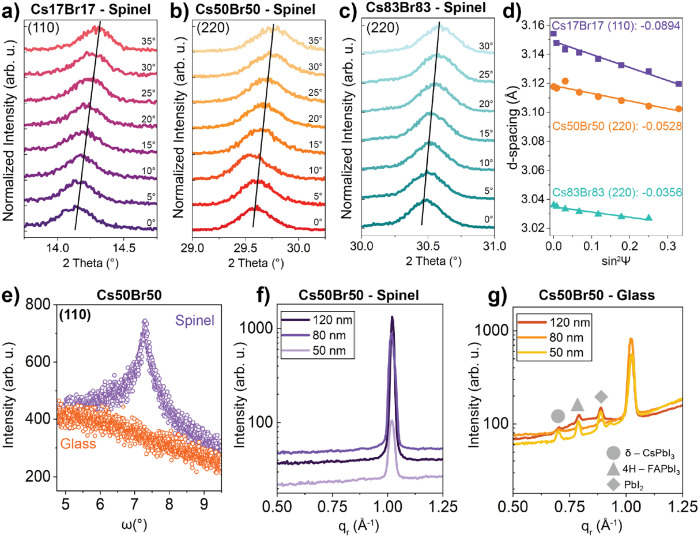
Depth- and thickness-dependent structural characterization. GIXRD measurements show MgAl_2_O_4_ induces compressive strain via peak shift to higher angles with increasing ψ (moving from out-of-plane toward in-plane) for (a) Cs17Br17 (110), (b) Cs50Br50 (220), (c) Cs83Br83 (220), (d) plots of d-spacing vs *sin* ^2^ *ψ*. The slope of peak position vs *sin* ^2^ *ψ* quantifies the magnitude of compressive strain, which decreases with decreasing δ, supporting mismatch-dependent lattice coupling. (e) Rocking curve measurements of the (110) LHP peak from 80 nm films on MgAl_2_O_4_ (spinel, purple) and glass (orange), indicating orientation induced by the substrate. Thickness-dependent out-of-plane sector averages around *q_r_* = 0 Å^–1^, plotted in log-scale to clearly visualize all phases, show that LHP films on (f) MgAl_2_O_4_ (spinel) are phase pure, while films on (g) glass have secondary phases as shown by peaks below *q_r_* = 1 Å^–1^.

To further examine polycrystalline thin-film orientation, rocking curve measurements of the (110) (2θ = 14^◦^) peak for Cs50Br50 thin films fabricated on spinel oxides and glass are shown in Figure 3e. Rocking curve measurements hold the detector position constant at a Bragg condition while varying ω to produce a bell curve of the distribution of planes as a function of sample tilt parallel to the X-ray beam.^78^ A heteroepitaxial film, like the LHP on spinel oxide shown in Figure 3a–e, will have a Gaussian-type curve, while a randomly oriented film will have a constant intensity as a function of ω (LHP on glass in Figure 3e).^78,79^ The rocking curve FWHM for the LHP on spinel oxide film is 0.537 ^◦^, which is a bit larger than in an epitaxial single crystal^80^ but similar to that reported in other LHP works that discuss heteroepitaxy.^44,81–83^ This further supports the role of the spinel oxide substrate in facilitating oriented, lattice-coupled, heteroepitaxial film growth. Structural characterization demonstrates that MgAl_2_O_4_ facilitates the growth of compressively strained, phase-pure LHP thin films across a wide range of compositions and δs, which are prone to secondary phases and low crystallinity when fabricated on glass. Cs50Br50 shows the largest differences in secondary phases and polycrystalline thin-film orientation between thin films grown on glass and MgAl_2_O_4_ substrates and thus is the composition chosen for deeper examination throughout the rest of this work.

To further investigate how far the effects of modifying the bottom interface propagate, we varied the precursor solution concentrations to change the final film thicknesses. Preliminary experiments were conducted with 0.6 M precursor solutions; 0.8 M and 0.4 M precursor solutions were added to expand the range of thicknesses to 50–130 nm. For Cs17Br17, films were also made with 1.2 M solutions to replicate device conditions (Figure S12). Film thicknesses measured using profilometry are shown in Table S3. Film thicknesses obtained on glass and MgAl_2_O_4_ are similar; measurements were taken in numerous locations with full coverage. Out-of-plane sector averages (*qr* = 0 Å^–1^, δ = ±30^◦^) from 2D GIWAXS patterns (Figure 3f,g) to characterize small amounts of oriented secondary phases show that, regardless of film thickness, fabricating films on spinel oxides prevents formation of secondary phases (no peaks below *q*_*r*_ = 1 Å^–1^). In contrast, films on glass have peaks that can be attributed to undesirable secondary phases (δ-CsPbI_3_, PbI_2_, and 4H-FAPbI_3_).^16,24,59–61^ Similar improvements in phase purity, regardless of film thickness, are also present in Cs17Br17 and Cs83Br83, with full 2D patterns of all compositions and thicknesses with replicates shown in Figures S13–S19. This demonstrates that, within the range of examined thicknesses, MgAl_2_O_4_ facilitates the growth of a phase-pure, crystalline LHP compared to films on glass that contain undesirable secondary phases.

### Lattice Coupling Homogenizes Intrinsic Phase Segregation

In addition to inducing beneficial compressive strain, coupling between the spinel oxide substrate and the LHP thin film should reduce defects at the bottom interface and result in improved overall film quality. To assess the effects of lattice coupling on optical properties and intrinsic phase segregation, we used cathodoluminescence-scanning electron microscopy (CL-SEM) on 80 nm Cs50Br50 LHP films. In CL-SEM, the electron beam used to take the SEM image simultaneously causes cathodoluminescence, the emission from which can be collected and separated into component wavelengths. Each component wavelength can then be used to create a high-resolution, correlative map of the emission and morphology. Our past works and others establish this technique for characterizing secondary phases and intrinsic phase segregation in LHPs.^16,84–86^ For example, PbI_2_ can be characterized by its distinctive CL emission around 505 nm, while hexagonal FAPbI_3_ phases can be identified by broad emission between 600 and 700 nm.^16,84,87^ Yellow overlays in Figure 4a,b show areas on the SEM image characterized as PbI_2_ from the 505 nm emission maps (Figure S20). The lack of overlays on MgAl_2_O_4_ (spinel) support structural characterization of suppressed secondary phase formation with microscale optical characterization. We attribute the reduction in secondary phase formation to reduced defect density due to templated crystallization at the bottom interface.^88^

**Figure 4:**
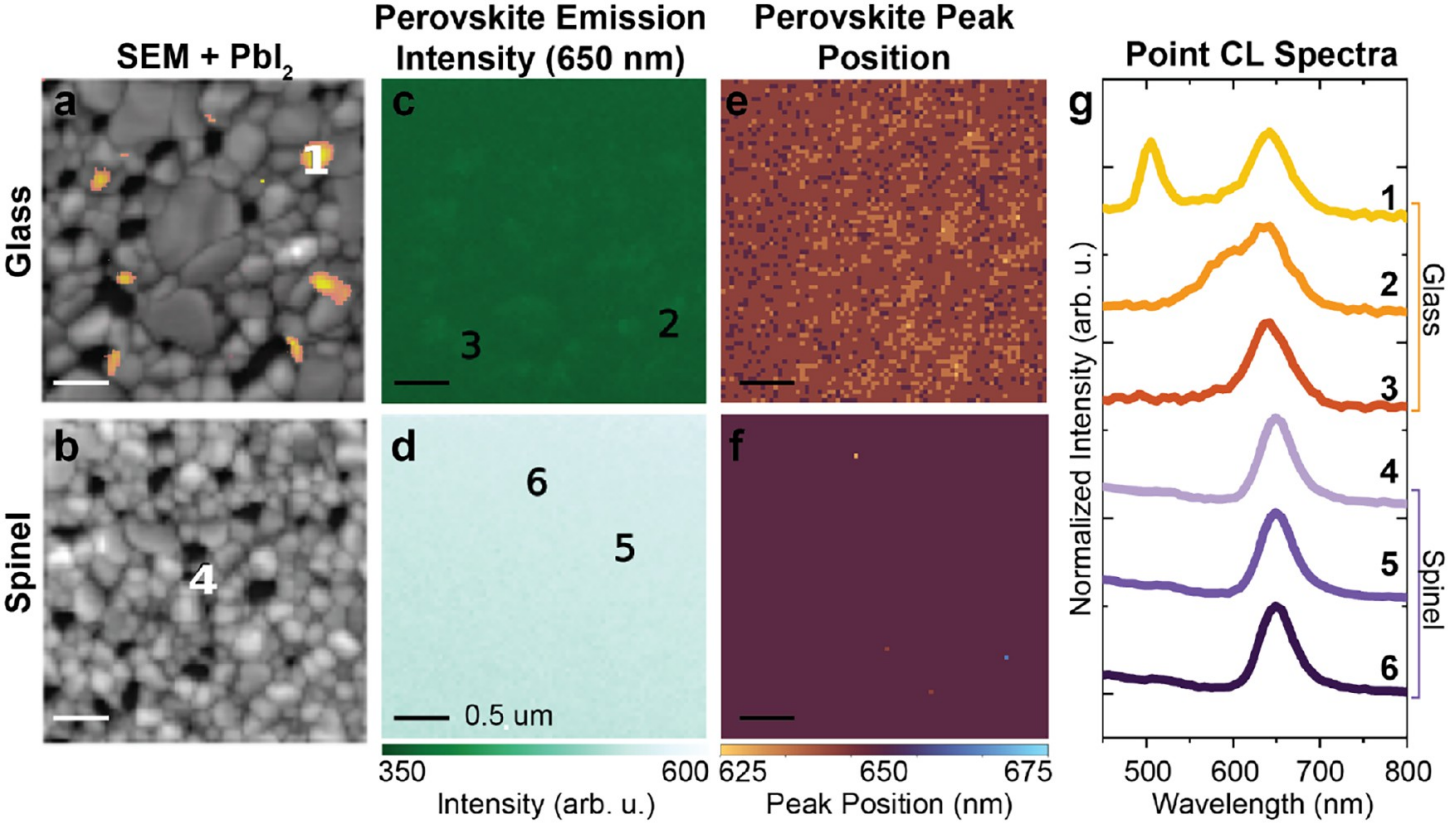
Spinel oxides improve and homogenize optical properties. (a,b) SEM images with extracted PbI_2_ overlays show that fabricating films on MgAl_2_O_4_ (spinel) reduces PbI_2_. Scale bar = 0.5 μm. (c,d) CL intensity maps of characteristic perovskite emission (650 nm). (e,f) Maps of perovskite emission peak position between 625 and 675 nm. (g) Normalized point CL spectra at representative points labeled in a–d: glass (orange) and MgAl_2_O_4_ (spinel, purple).

To understand the effect of lattice coupling on intrinsic phase segregation and optical properties of the LHP, we can plot the characteristic perovskite emission. From the perovskite emission (650 nm) maps, we can see that fabricating films on MgAl_2_O_4_ improves the optical properties, as CL intensity is higher and more homogeneous than for films on glass (Figure 4c,d). This is likely due to templated crystallization at the bottom interface, which can reduce defects and thus suppresses nonradiative recombination.^36^ The precise emission peak position can be used to examine intrinsic phase segregation, based on changes in the bandgap due tothe local perovskite composition.^15^ Throughout this work, we refer to intrinsic phase segregation of halides or cations that occurs during crystallization, rather than photoinduced halide segregation. The perovskite emission peak position (between 625 and 675 nm) is plotted in Figure 4e,f. On glass (Figure 4e), there is significant variation in the LHP emission peak position, which can be attributed changes in the local bandgap due to intrinsic phase segregation.^18^ It is important to note here that phase segregation often presents only as a longer wavelength iodide-rich phase, as CL from the wider bandgap bromide-rich phase can be quenched by the narrow bandgap iodide-rich phase via charge funneling.^89,90^ The distribution of peak position thus should not be considered to represent the exact compositional distribution, but rather as evidence that compositional variation is present. Intrinsic phase segregation is largely reduced on MgAl_2_O_4_, as seen by the homogeneous emission peak position map in Figure 4f. This is likely due to templated crystallization reducing defects and substrate-induced compressive strain, which has been demonstrated to limit the ion migration required for phase segregation.^52,91^ Figure 4g shows representative CL point spectra. On glass, there are regions of PbI_2_ (point 1: secondary peak around 505 nm), phase segregation (point 2: bromide-rich emission appears as a broad secondary peak), and pristine perovskite (point 3).^14,16,86^ All point spectra on MgAl_2_O_4_ (spinel) show homogeneous characteristic perovskite emission, with a small shoulder below 550 nm due to the broad CL of MgAl_2_O_4_.^92,93^ Via the increased emission intensity and homogeneous emission peak positions, we confirm that spinel oxides improve optical properties and reduce intrinsic phase segregation, likely by improving film quality at the bottom interface and inducing compressive strain. We see the same trends in the Cs83Br83 system, further supporting the beneficial effects of lattice coupling across a range of δs and chemistries (Figure S21).

To examine phase segregation through the film bulk, we used X-ray fluorescence (XRF) to map the elemental distribution of the cations and halides (Figure 5). The high-energy X-rays used for XRF (14.5 keV) probe the entire film, while CL-SEM focuses primarily on the surface.^71,94^ XRF maps of elemental ratios are used to analyze the compositional distribution in perovskite thin films while accounting for local thickness variations.^95^ I:Br elemental ratios of LHP thin films of varying thicknesses fabricated on glass and MgAl_2_O_4_ are shown in Figure 5a,b. Measurements were taken in areas with full coverage. Across film thicknesses, LHP films on glass exhibit significant heterogeneities in halide distribution, as shown by variations in I:Br ratio (Figure 5a). In contrast, LHP films on MgAl_2_O_4_ display a much more homogeneous I:Br ratio. This homogenization of local compositional changes shows that MgAl_2_O_4_ reduces the intrinsic halide segregation through the film bulk. The same suppression of phase segregation can be seen relative to both the A-site cation and Pb; spinel oxide substrates reduce local compositional changes in both Br:Pb (Figure 5b) and Cs:Pb elemental ratios (Figure S22).^96,97^ To quantify variation in elemental ratios, we calculated the coefficient of variation (standard deviation/mean) for each condition. A higher coefficient of variation indicates a greater distribution of the elemental ratios. For I:Br (Figure 5c), Br:Pb (Figure 5d), and Cs:Pb (Figure S22), films on glass have higher coefficients of variation than LHPs fabricated on spinel oxides. This shows that lattice coupling with MgAl_2_O_4_ quantitatively reduces phase segregation through the entire film, not just at the bottom interface where bonding directly occurs.

**Figure 5:**
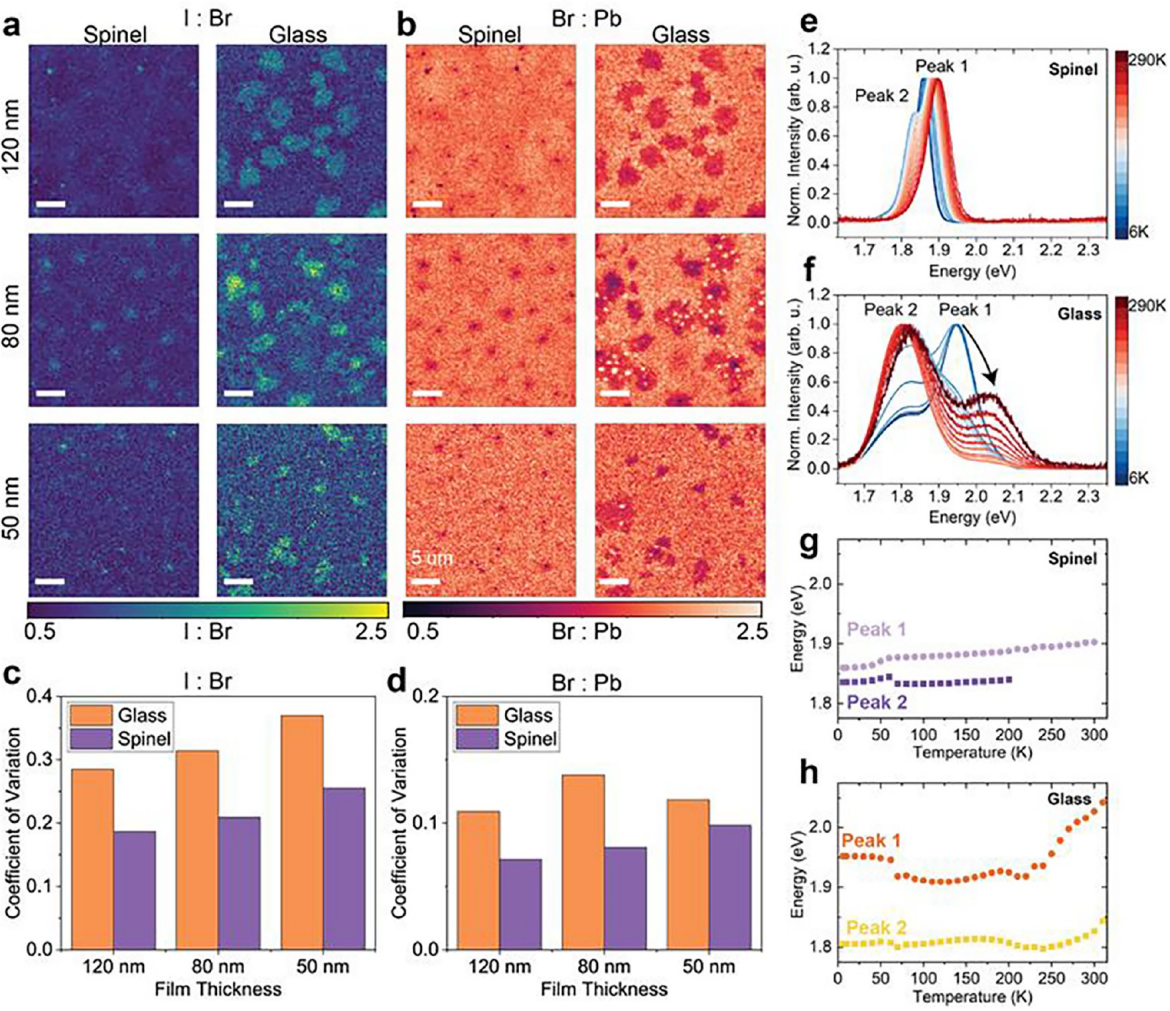
Lattice coupling suppresses phase segregation through film bulk. (a) I:Br and (b) Br:Pb elemental ratios from XRF mapping on MgAl_2_O_4_ (spinel) and glass. Coefficient of variation (standard deviation/mean) of elemental ratios on MgAl_2_O_4_ (spinel) and glass: (c) I:Br, (d) Br:Pb. Normalized temperature-dependent photoluminescence spectroscopy (PL) of Cs50Br50 thin films fabricated on (e) MgAl_2_O_4_ (spinel) and (f) glass. Fitted PL peak positions from e,f as a function of temperature of Cs50Br50 thin films fabricated on (g) MgAl_2_O_4_ (spinel) and (h) glass.

To check the conclusions of XRF against a surface-only elemental technique, we measured X-ray photoelectron spectroscopy (XPS) and calculated the same elemental ratios. XRF penetrates, and thus provides compositional information on, the whole bulk, while XPS measures approximately only the top 10 nm.^200^ If there were a spinel oxide-induced compositional gradient, then there would be significant differences in elemental ratios measured by XRF and XPS. Br:Pb elemental ratios are similar between the two techniques, indicating that there is no compositional gradient and further supporting that substrate-induced lattice coupling influences crystallization of the entire film, not just the bottom interface (Figure S23).

We then performed cryogenic-temperature-dependent photoluminescence (PL) measurements to determine whether the films consist of intrinsically segregated domains or a single mixed perovskite phase that undergoes dynamic, temperature-driven segregation processes. We first confirmed that the emission is independent of the excitation power at room temperature and after cooling to 6 K (Figure S24). The LHP films on MgAl_2_O_4_ have a dominant emission peak around 1.9 eV that redshifts as temperature decreases, along with a weaker, lower-energy feature evident at low temperatures (Figure 5e). This fine-structure splitting at low temperatures is on the order of meV and is typical of LHPs.^98^ In contrast, the films fabricated on glass exhibit a markedly different temperature dependence of the emission (Figure 5f). Unnormalized plots are shown in Figure S24. At room temperature, two emission peaks are visible: a lower-energy peak at 1.8 eV and a shoulder at 2.05 eV, separated by an energy of more than 0.2 eV from the main peak. This shoulder increases in intensity as the temperature is reduced, eventually surpassing the lower-energy emission intensity. Plots of the specific peak positions are shown in Figure 5g,h. This indicates the presence of multiple segregated phases on glass, with charge transfer between these phases that is kinetically activated with temperature, corroborating the bulk phase segregation seen on glass and the lack thereof on spinel oxides observed with CL-SEM (Figure 4) and XRF (Figure 5a–d).^99,100^ Additionally, peaks from films fabricated on glass have significant broadening compared to those on MgAl_2_O_4_; we attribute this to the reduction of defects and grain boundary effects as a result of lattice coupling.^101^

### Lattice Coupling with Spinel Oxides Improves Stability

To elucidate the mechanism by which spinel oxides enhance phase purity and suppress phase segregation in LHP thin films, we performed DFT calculations on model CsPbI_3_/MgAl_2_O_4_ interfaces. Considering different surface terminations for both materials, we found that the two interfacial configurations with Mg termination are the most energetically favorable (Figure 6a,b). At these interfaces, I–Mg bonds form between the spinel oxide and LHP, with negative interface formation energies of approximately −2.9 eV for both PbI_2_- and CsI-terminated CsPbI_3_ surfaces. This suggests that either cation-halide- or lead-halide-terminated surfaces can bond effectively with the spinel oxide substrate to template thin film growth. As the lattice parameter of all LHP compositions is slightly larger than that of the MgAl_2_O_4_, this bonding induces compressive strain in the model system, as supported by structural characterization (Figure 3), which has been shown to increase the barrier for ion migration.^52,73^

**Figure 6:**
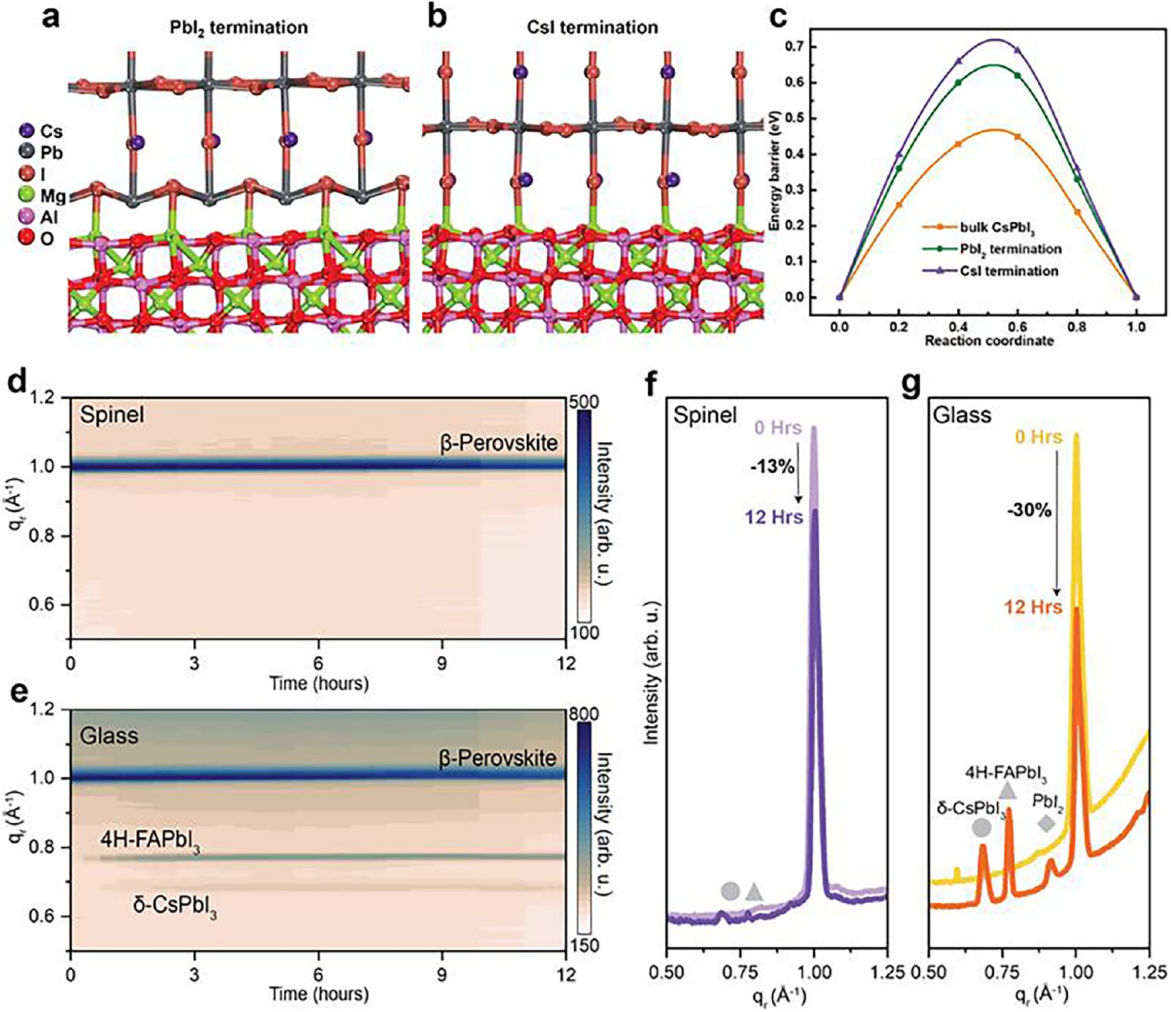
Lattice coupling with spinel oxides improves LHP stability. DFT simulations of the of a) PbI_2_ termination and b) CsI terminations for MgAl_2_O_4_–CsPbI_3_ lattice matching. c) iodide diffusion barriers from NEB calculations for pristine CsPbI_3_ and lattice matched interfaces. In situ GIWAXS under 100% relative humidity of 80 nm Cs50Br50 LHP films on d) MgAl_2_O_4_ (spinel) and (e) glass with continuous measurements over 12 h (color mapped circular averages). Circular averages at 0 and 12 h on f) MgAl_2_O_4_ (spinel) and g) glass show significant secondary phase formation on glass and a 30% reduction of perovskite peak intensity compared to improved stability for films on MgAl_2_O_4_.

To examine defect formation and ion migration in lattice-matched systems, we calculated the formation energy of iodide vacancies at the MgAl_2_O_4_-CsPbI_3_ interfaces and compared them with those in bulk CsPbI_3_. We found that the vacancy formation energy increases from 1.79 eV in the bulk to 2.26 and 2.41 eV for the lattice-matched PbI_2_- and CsI-terminated interfaces, respectively. Higher formation energies suggest that defect formation is thermodynamically less favorable at the lattice-matched interface, and thus defect-driven processes like ion migration and phase segregation are likely suppressed. We further evaluated the kinetic aspect of ion migration by calculating iodide diffusion barriers using the Nudged Elastic Band (NEB) method in the bulk CsPbI_3_ versus at the spinel oxide-LHP interfaces (Figure 6c). The diffusion barrier for iodide in bulk CsPbI_3_ was found to be 0.45 eV, consistent with previous report,^102^ but increases to 0.62 and 0.69 eV for the modified PbI_2_- and CsI-terminated interfaces, respectively. This enhancement can be attributed to the compressive strain imposed by bonding with the MgAl_2_O_4_ substrate, which is known to increase halide migration barriers.^52,103^ The combination of higher vacancy formation energy and higher diffusion barrier suggests that the spinel oxide-perovskite interface stabilizes the LHP by inducing compressive strain, which reduces halide ion mobility and thereby mitigates phase segregation. To verify the generality of this mechanism, we also performed analogous calculations for CsPbBr_3_/MgAl_2_O_4_ interfaces (Figures S25 and S26). A similar trend was observed, though the magnitude of the increase in vacancy formation energy and diffusion barrier was smaller, consistent with the reduced lattice mismatch between CsPbBr_3_ and MgAl_2_O_4_ that results in less compressive strain. Direct comparison of the two model systems showing that beneficial effects scale with increasing lattice mismatch is presented in Figure S26. This suggests that compressive strain, plays a key role in limiting ion migration, which may be more beneficial than achieving perfect lattice mismatch.Though interfacial chemistry may also play a role in the changes seen after fabricating on spinel oxides, the scaling of migration barriers, compressive strain, and lattice mismatch supports the conclusion that lattice coupling plays a significant role in governing crystallization. Overall, these DFT results provide a fundamental understanding of how spinel oxides stabilize LHP films by inducing compressive strain that impedes halide ion migration and thus suppresses defect-mediated phase segregation and degradation. Decreasing δ should increase the coherency of the bottom interface and further reduce defects, but would also reduce the beneficial effects of compressive strain, indicating a need to balance the two parameters. We hypothesize that the role of compressive strain is more beneficial than the reduction of defects, based on the improvements in optoelectronic properties and chemistry shown here, and encourage a focus on prioritizing device integration rather than achieving perfect lattice matching.

If spinel oxide/LHP lattice coupling reduces halide mobility and defect densities, this should also translate to improved stability, as ion migration plays a key role in environmental degradation.^1,20,28^ We used in situ GIWAXS under 100% relative humidity on 80 nm Cs50Br50 films for 12 h (Figure 6d,e) to track how lattice coupling affects phase transformations. LHPs on MgAl_2_O_4_ (spinel) substrates showed no major structural changes over 12 h in 100% humidity (Figure 6d,f) as indicated by the relatively constant β-perovskite peak around *q*_*r*_ = 1 Å^–1^ and no significant appearance of new peaks related to secondary phase formation. In contrast, films fabricated on glass show quick growth of secondary phases (δ-CsPbI_3_ and 4H-FAPbI_3_) as evidenced by the appearance of peaks below *q*_*r*_ = 1Å^–1^ (Figure 6e,g), which is supported by known humidity degradation mechanisms.^97^ Quantifying peak intensity changes from initial and final circular averages shows that the LHP thin film on spinel oxide retains 87% of its perovskite peak intensity intensity after 12 h in 100% relative humidity. Films fabricated on glass retain only 70% of their perovskite peak intensity and see significant formation of secondary phases (Figure 6f,g and Figure S27). LHPs on spinel oxides demonstrate impressive long-term stability under 100% relative humidity, in agreement with reduced defect formation and ion migration as suggested by DFT (Figure 6) and heteroepitaxial growth theory.^46^ This is further evidence that lattice coupling stabilizes the film bulk, not just the bottom interface, both by reducing defects through templated crystallization and by inducing compressive strain, which reduces halide mobility and subsequent defect-driven degradation processes. We also assessed stability under temperature and light stressing (Figures S28 and S29). There are no significant changes in thermal stability as a result of lattice coupling (Figure S28). Instead, thermal stability increases with increasing Cs, which aligns with literature mechanisms that attribute thermal instability to the loss of organic and halides from the top surface.^16,104^ In situ photoluminescence measurements show improved stability under illumination for LHP films on spinel oxides, supporting bulk improvements in film quality after lattice coupling (Figure S29).

## Conclusions

We used MgAl_2_O_4_ to facilitate epitaxy-inspired, lattice-coupled LHP thin-film crystallization and growth over a range of compositions, δs, and thicknesses. Spinel oxides facilitate compressively strained, phase-pure crystalline film growth, as shown by GIWAXS and GIXRD measurements. Comprehensive morphological, optical, and elemental characterization shows that spinel oxides reduce phase transformations and intrisic phase segregation over the film bulk, likely by improving film quality at the bottom interface and inducing compressive strain. DFT calculations support both the thermodynamic feasibility of MgAl_2_O_4_–LHP coupling and the suppression of defect formation and subsequent migration due to mismatch-dependent compressive strain. Crucially, we directly visualize a reduction in intrinsic phase segregation through the bulk, emphasizing the importance of the bottom interface in determining overall film quality. Spinel oxides also improve long-term stability under humidity through improvements in structural phase purity and likely reductions in defect density and halide ion mobility. This expands the scope and understanding of lattice coupling and strain engineering to improving fundamental material quality, which translates to improved performance and efficiency in optoelectronic devices. While integration into a device is outside the scope of this work, we establish the benefits that would accompany the integration of a lattice-matched spinel oxide layer into a full solar cell. Future work should explore integrating other spinel oxides with matching lattice parameters and appropriate band alignments for CTLs, such as NiCo_2_O_4_, into devices by focusing on depositing a highly crystalline thin layer in a full device stack.^50,54^ Integrating a highly crystalline NiCo_2_O_4_ layer (*a* = 8.24 Å) as a hole transport layer in an inverted device architecture, for example, could template crystallization for oriented, phase-pure film growth with suppressed phase segregation and improved environmental stability. Vapor deposition of MgAl_2_O_4_ could also be used to deposit a very thin layer to template growth with minimal interference with charge transfer in a PSC device stack.^105,106^ This work establishes lattice matching as a technique to stabilize difficult bromide-rich compositions against intrinsic phase segregation and degradation for multijunction applications and confirms spinel oxides as a promising, novel materials family for applications in LHPs and PSCs.

## Methods

### Thin-Film Fabrication

1 cm × 1 cm soda lime glass or (100) polished MgAl_2_O_4_ substrates (MTI Corporation) were cleaned by sonicating for 15 min in deionized water, 30 min in acetone, and 30 min in isopropanol (IPA), and dried with a nitrogen gun after the deionized water and IPA steps. Substrates were then cleaned with the UV-ozone cleaner for 15 min directly before spincoating. All solution preparation, spincoating, and annealing were carried out inside a nitrogen glovebox. Stoichiometric Cs-FA Pb I–Br precursor solutions of the appropriate molarities were prepared by by combining FAI (Greatcell Solar), CsI (Sigma-Aldrich), PbI_2_ (Tokyo Chemical Industry - TCI), PbBr_2_ (TCI), and CsBr (Sigma–Aldrich) (only for Cs83Br83) in appropriate molar ratios in one vial and dissolving them in dimethyl sulfoxide (DMSO) (Sigma–Aldrich) by vortexing. 1.2M Cs17Br17 films to replicate device conditions were made in 2:1 dimethyformamide­(DMF)/DMSO volumetric ratios. 60 μL of perovskite precursor solution was deposited statically, and then spin-coated with a two-step process (1000 rpm for 10 s, 6000 rpm for 20 s). 125 μL of chlorobenzene (Sigma–Aldrich) was dynamically spin-coated 5 s before the end of the spin-coating. Thin films were then annealed at 150 ^◦^C for 10 min.

### Characterization

Thin film materials characterization was performed at Georgia Tech in the Institute for Matter and Systems Materials Characterization Facilities. SEM images were taken on a Hitachi 8230 SEM. Rocking curve and GIXRD *sin* ^2^ *ψ* XRD measurements were taken on a Rigaku Smartlab XE with a Copper Kα source in the parallel beam configuration (details in Supplementary Note 1). A height alignment was performed for each sample prior to measurement to ensure no peak shift was due to slight changes in sample height or thin film thickness. X-ray photoelectron spectroscopy measurements were taken on a Thermo Scientific K-Alpha system equipped with a monochromatic Al Kα X-ray source (hv = 1486.6 eV). The incident X-ray beam was aligned at 60^◦^ relative to the sample normal, while photoelectrons were collected at 0^◦^ emission angle. All spectra were acquired under high vacuum conditions, with the chamber pressure maintained below 1 × 10^–7^ Torr. Both survey and high-resolution elemental scans were obtained. Survey spectra were averaged over two measurements with 200 eV pass energy, 50 ms dwell time, and 0.1 eV step size. High-resolution scans were averaged over 20 measurements for C 1s and 10 measurements for Br 3d, Pb 4f, and I 3d. Peak fitting was conducted using the Thermo Scientific Avantage Data System.

### Photoluminescence

#### Cryogenic Temperature-Dependent Photoluminescence

Photoluminescence measurements were performed by illuminating the sample with a 485 nm continuous-wave laser at a power of 5.97 mW cm^–2^, generated by a Melles Griot 25-LAP-431–208 ion-laser system. The sample was mounted inside a Montana Instruments closed-cycle helium gas-exchange cryostat. Measurements were collected in steps during the warm-up cycle after cooling to 6 K. The incident power was measured using a calibrated photodiode, and the photoluminescence was collected by a set of lenses and directed to a spectrometer.

#### Photoluminescence with Illumination

Samples were held under 0.5 Sun illumination in a nitrogen glovebox and measured periodically on a homemade photoluminescence setup. A ThorLabs 405 nm LED laser was used as a light source, with the power adjusted to 2.5 mW using an attenuator wheel and an acquisition time of 0.5 s.

### Grazing Incidence Wide-Angle X-ray Scattering

GIWAXS measurements were carried out at Brookhaven National Laboratory at the NSLS-II on Beamline 11-BM. Ex situ measurements were carried out under low vacuum. The X-ray beam had an energy of 13.5 keV, with a footprint of 0.2 mm × 0.05 mm. The samples were irradiated for 10 s with incident angles (α) of 0.1^◦^ (surface) and 0.5^◦^ (bulk). Beam divergence was 1 mrad, and energy resolution was 0.7%. The data were analyzed using the SciAnalysis Python package provided by the beamline. In situ humidity measurements were carried out in 100% relative humidity with air as the carrier gas. A measurement was taken before starting the humidity flow, and once the humidity flow began, measurements were taken continuously over a 12 h time period. In situ temperature measurements were taken on a thermal stage. Samples were first measured at room temperature. Then, the temperature was ramped to 75 ^◦^C and samples were realigned to account for thermal expansion before measuring, which took approximately 45 min. The temperature was then increased to 150 ^◦^C and the same procedure was followed.

### Cathodoluminescence SEM

CL-SEM measurements were carried out at the Center for Nanophase Materials Sciences at the Oak Ridge National Laboratory. An FEI Quattro environmental SEM instrument with a Delmic Sparc CL collection module was used, equipped with a parabolic mirror to collect the CL signals from the film after excitation. An electron beam with an acceleration voltage of 5 kV and a beam current of 14 pA was passed through a hole in the parabolic mirror for sample excitation. The CL signal collection acquisition time was 350 ms per spectrum, with a pixel size of 40 nm. All measurements were conducted in a low-vacuum environment of 0.35 Torr H_2_O vapor to mitigate sample charging.^16,84^ Data was then processed on Google Colab using Python 3.6 and the scikit-learn 0.22.1 library, utilizing notebooks created by Sergei Kalinin and Jonghee Yang.^16,84,107^ Perovskite peak position maps were created by finding the maximum position of peaks between 625-675 nm for Cs50Br50 and 515–575 nm for Cs83Br83.

### X-ray Fluorescence Mapping

XRF measurements were carried out at the Advanced Photon Source at Argonne National Laboratory on beamline 2-ID-D. The synchrotron X-ray energy was 14 keV, with a dwell time of 50 ms and a step size of 0.25 μm. We used the CsBr and PbI_2_ standards to establish the proper branching ratio of Cs_L and I_L XRF emission. The XRF-MAPS software was used for data analysis and spectrum fitting to deconvolute overlapping peaks and background from the fluorescence data.^108^

### Calculations

Spin-polarized density functional theory (DFT) calculations were performed using the Vienna Ab Initio Simulations Package (VASP).^109^ The electron exchange–correlation was represented by the functional of Perdew, Burke, and Ernzerhof (PBE) within the generalized gradient approximation (GGA).^110^ The ion–electron interaction was described with the projector augmented wave (PAW) method.^111^ A cutoff energy of 500 eV was used for the plane-wave basis set. The energies were converged with a 1 × 10^–5^ eV tolerance, and the forces were optimized to within 0.025 eV/Å. The climbing-image nudged elastic band method, implemented in VASP, was used to determine the energy barriers.^112^ The transition states were obtained by relaxing the forces below 0.05 eV/Å.

## Supplementary Material



## Notes

Supporting Information includes additional GIXRD methods, GIWAXS and GIXRD patterns, XRF maps, X-ray photoelectron spectroscopy, CL-SEM maps, calculations, and raw data from literature included in figures (PDF)
