# Supplementary material for: Suppressing Phase Segregation and Improving Stability in Mixed-Halide Perovskites through Spinel Oxide-Directed Epitaxy
Source: J Am Chem Soc. 2026 Jun 15;148(25):26076–91. doi: 10.1021/jacs.6c04846 (PMC13339156; doi:10.1021/jacs.6c04846)
Supplement: Supplementary file 1 [file ja6c04846_si_001.pdf]

# Supplementary Information: Suppressing Phase Segregation and Improving Stability in Mixed Halide Perovskites through Spinel Oxide-Directed Epitaxy

Diana K. LaFollette[1], Martin Gomez-Dominguez[1], Kunal Datta[1], Amanda Conde del Moral[1], Seungjun Cha[1], Jack Lawton[1], Ruipeng Li[2], Benjamin Lawrie[3,4], Yanqi Luo[5], Carlo A.R. Perini[1], Guoxiang Hu[1,6], Juan-Pablo Correa-Baena[1,6*]

[1*]School of Materials Science and Engineering, Georgia Institute of Technology, Atlanta, 30332, Georgia, United States.
[2]National Synchrotron Light Source II, Brookhaven National Laboratory, Upton, 11973, New York, United States.
[3]Center for Nanophase Materials Science, Oak Ridge National Laboratory, Oak Ridge, 37831, Tennessee, United States.
[4]Materials Science and Technology Division, Oak Ridge National Laboratory, Oak Ridge, 37831, Tennessee, United States.
[5]Advanced Photon Source, Argonne National Laboratory, Lemont, 60439, Illinois, United States.
[6]School of Chemistry and Biochemistry, Georgia Institute of Technology, Atlanta, 30332, Georgia, United States .

*Corresponding author(s). E-mail(s): jpcorrea@gatech.edu;

# 1 Supplementary Material

**Table S1** Lattice Parameters (Å) of Cubic Spinel Oxides from Figure 1d and more. [1]

| | | | | | |
|---|---|---|---|---|---|
| SiFe2O4 | 7.96566 | NiRh2O4 | 8.34479 | MnRh2O4 | 8.50309 |
| SiNi2O4 | 8.07119 | ZnGa2O4 | 8.34986 | FeTi2O4 | 8.52699 |
| NiAl2O4 | 8.07785 | ZnCr2O4 | 8.35033 | MnTi2O4 | 8.57952 |
| MgAl2O4 | 8.13411 | GeMg2O4 | 8.35755 | TiFe2O4 | 8.74667 |
| ZnAl2O4 | 8.15475 | NiMn2O4 | 8.35788 | MgIn2O4 | 8.78157 |
| SnMg2O4 | 8.19491 | LiV2O4 | 8.36217 | HgCr2O4 | 8.78984 |
| SiMg2O4 | 8.19799 | GeCo2O4 | 8.37077 | MnIn2O4 | 8.88361 |
| SiCo2O4 | 8.21121 | FeGa2O4 | 8.37873 | MoNa2O4 | 9.15768 |
| SiZn2O4 | 8.21859 | MgFe2O4 | 8.38996 | WNa2O4 | 9.16011 |
| GeNi2O4 | 8.23075 | MgV2O4 | 8.39474 | MoAg2O4 | 9.34341 |
| MnAl2O4 | 8.23615 | MgRh2O4 | 8.40105 | | |
| RuCo2O4 | 8.24101 | MoFe2O4 | 8.41044 | | |
| NiCo2O4 | 8.24192 | ZnFe2O4 | 8.4106 | | |
| NiGa2O4 | 8.27295 | ZnV2O4 | 8.41539 | | |
| NiCr2O4 | 8.27342 | ZnRh2O4 | 8.4217 | | |
| TiMg2O4 | 8.28014 | MnGa2O4 | 8.43125 | | |
| MgCo2O4 | 8.29818 | MnCr2O4 | 8.43172 | | |
| PdZn2O4 | 8.3045 | FeV2O4 | 8.44426 | | |
| LiMn2O4 | 8.31161 | TiMn2O4 | 8.4468 | | |
| FeNi2O4 | 8.32012 | GeFe2O4 | 8.46254 | | |
| TiZn2O4 | 8.32572 | FeMn2O4 | 8.46366 | | |
| MgGa2O4 | 8.32922 | MgTi2O4 | 8.47748 | | |
| MgCr2O4 | 8.32968 | MnFe2O4 | 8.49199 | | |
| NiFe2O4 | 8.33369 | MnV2O4 | 8.49678 | | |

**Table S2** a and b lattice parameters (Å) shown in Figure 1d for CsFAPbIBr tetragonal LHP structures.[2]

| | **Pure I** | **83% I** | **66% I** | **50% I** | **33% I** | **17% I** | **Pure Br** |
|---|---|---|---|---|---|---|---|
| **Pure Cs** | 8.64 | 8.53 | 8.4 | 8.34 | 8.21 | 8.14 | 8.11 |
| **83% Cs** | 8.64 | 8.55 | 8.46 | 8.37 | 8.3 | 8.19 | 8.11 |
| **66% Cs** | 8.66 | 8.56 | 8.47 | 8.4 | 8.34 | 8.24 | 8.13 |
| **50% Cs** | 8.68 | 8.59 | 8.47 | 8.42 | 8.34 | 8.26 | 8.17 |
| **33% Cs** | 8.7 | 8.6 | 8.51 | 8.46 | 8.38 | 8.31 | 8.24 |
| **17% Cs** | 8.37 | 8.59 | 8.57 | 8.43 | 8.41 | 8.32 | 8.23 |
| **Pure FA** | 8.69 | 8.65 | 8.56 | 8.49 | 8.41 | 8.31 | 8.29 |

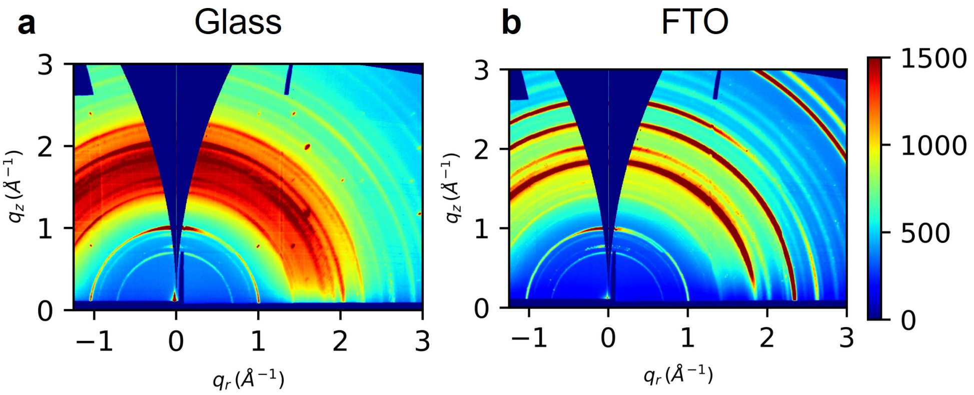


**Fig. S1** 2D GIWAXS patterns of Cs50Br50 on a) glass and b) glass/FTO demonstrating that the presence of a TCO does not fundamentally change crystallization. Glass is used for the rest of the paper to avoid influence of $SnO_2$ peaks.

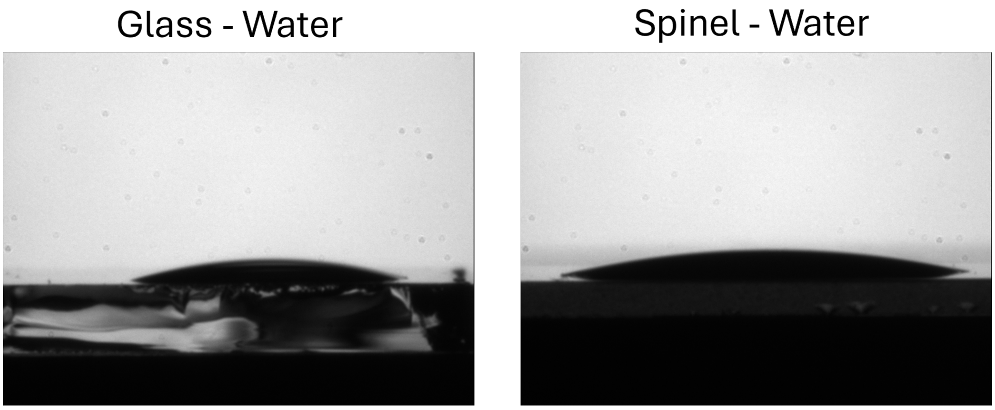


**Fig. S2** Contact angle images of water droplets on glass and $MgAl_2O_4$ show that both substrates are very wettable. This supports the effect of lattice matching in causing changes in crystallization, rather than substrate chemistry or surface energy. Measurements were conducted with 7.5 $\mu$L of water.

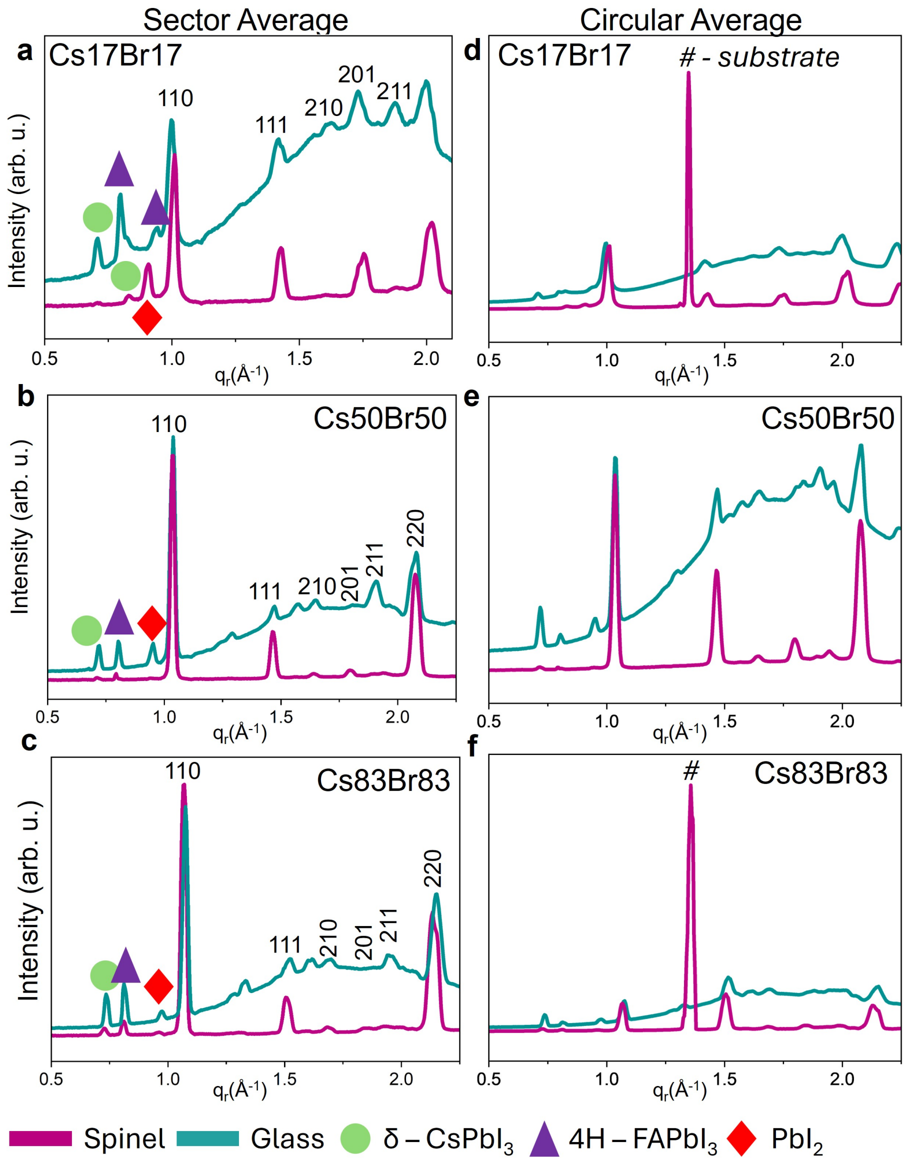


**Fig. S3** Peak Indexing of Sector Averages from Figure 2 for LHPs on $MgAl_2O_4$ (spinel, pink) and glass (teal) for a) Cs17Br17, b) Cs50Br50, and c) Cs83Br83. By using the sector average around $q_r = 0\,\text{Å}^{-1}$, we can omit the scattering from the $MgAl_2O_4$ (111). Tetragonal LHPs can be differentiated from cubic based upon the presence of the (210) and (211) peaks. Circular averages from Figure 2 with $MgAl_2O_4$ peaks (#) for d) Cs17Br17, e) Cs50Br50, and f) Cs83Br83.

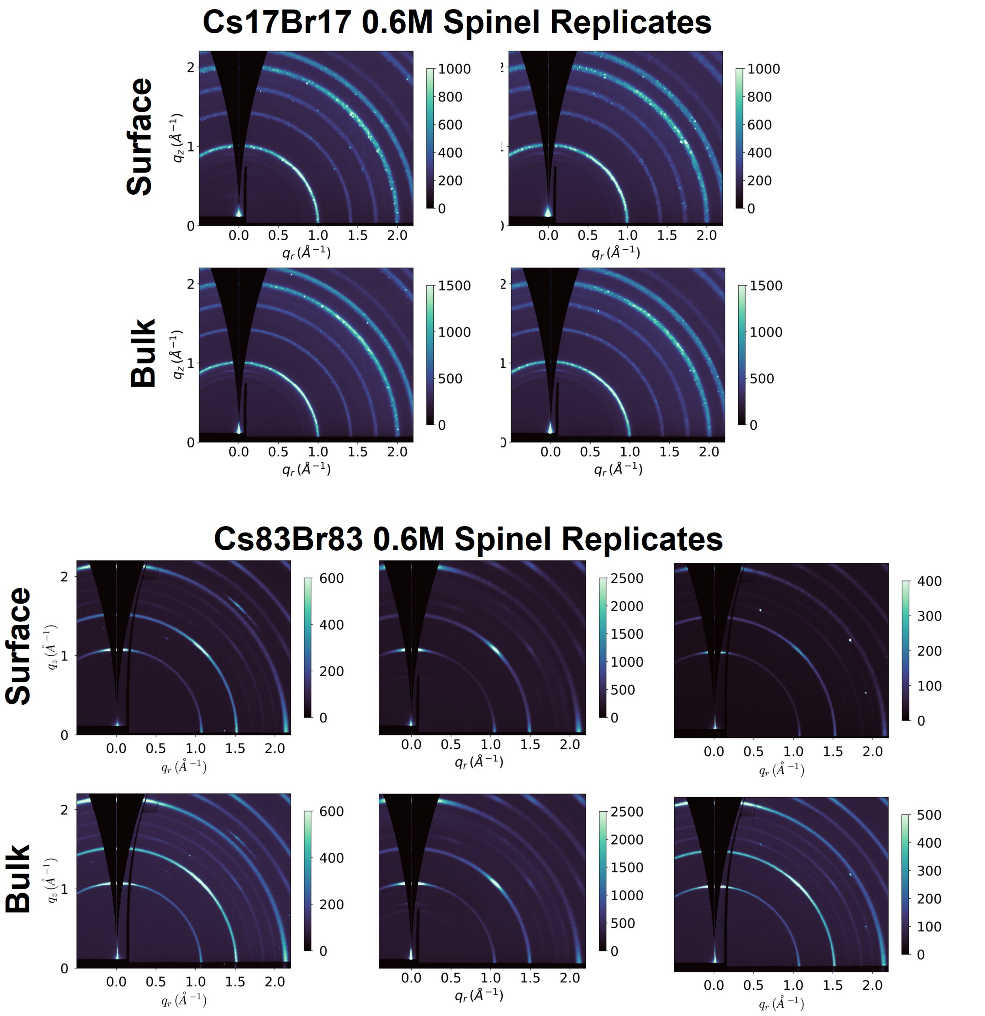


**Fig. S4** Spinel 0.6 M Reproducibility Check - Cs17Br17 and Cs83Br83. Replicates across samples and batches show same phases present, with only slight variations in intensity or orientation.

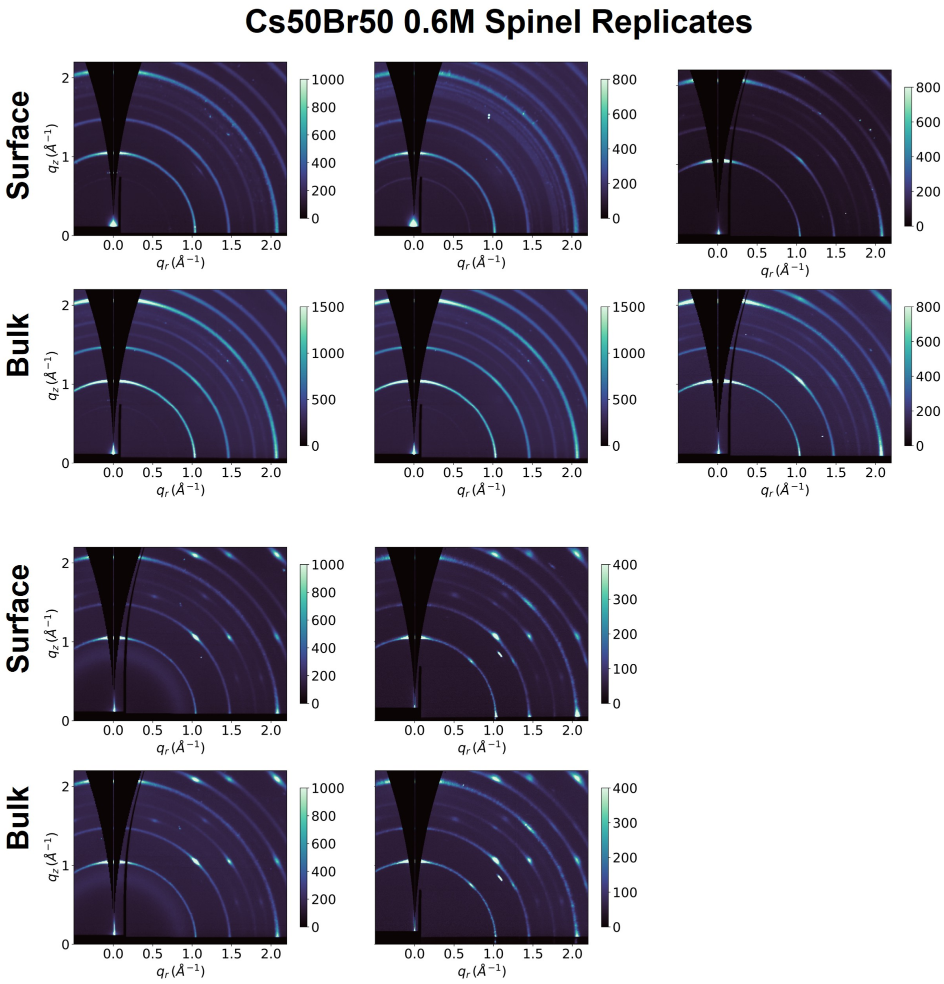


**Fig. S5** Spinel 0.6 M Reproducibility Check - Cs50Br50. Replicates across samples and batches show same secondary phases present, with only slight variations in intensity or orientation.

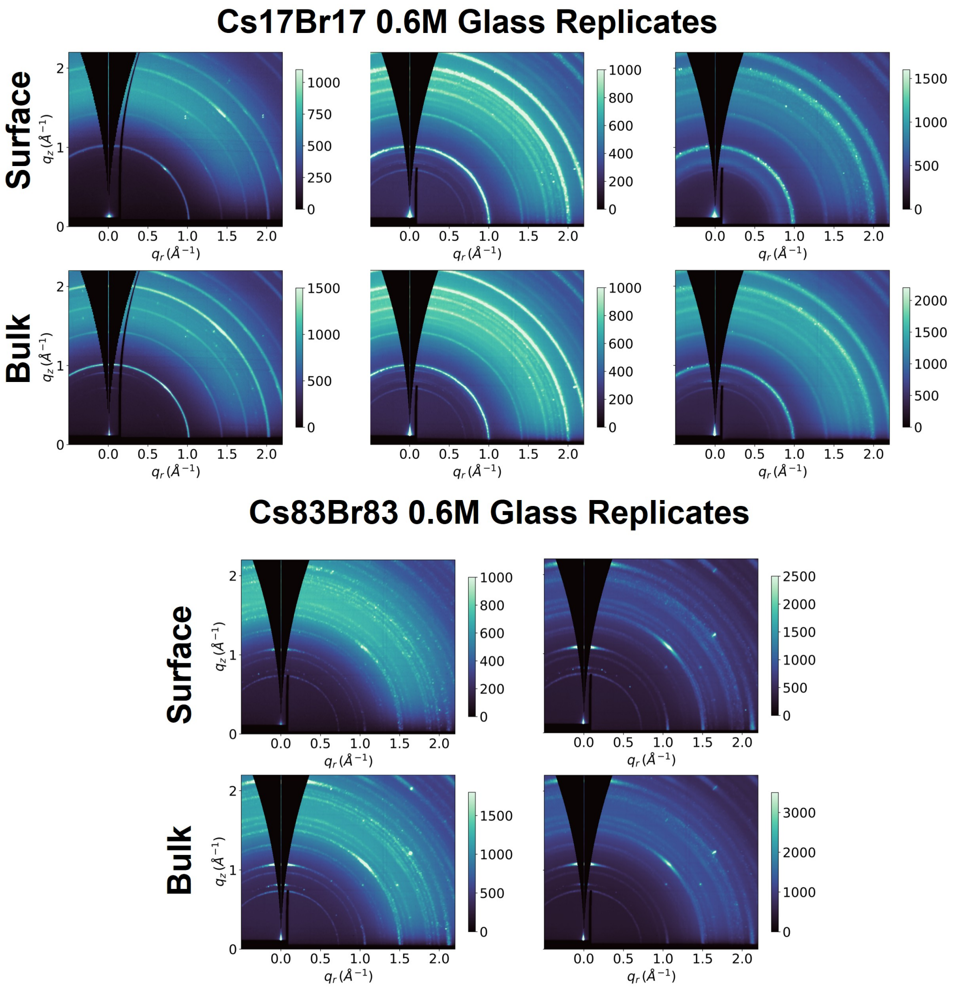


**Fig. S6** Glass 0.6 M Reproducibility Check - Cs17Br17 and Cs83Br83. Replicates across samples and batches show same secondary phases present, with only slight variations in intensity.

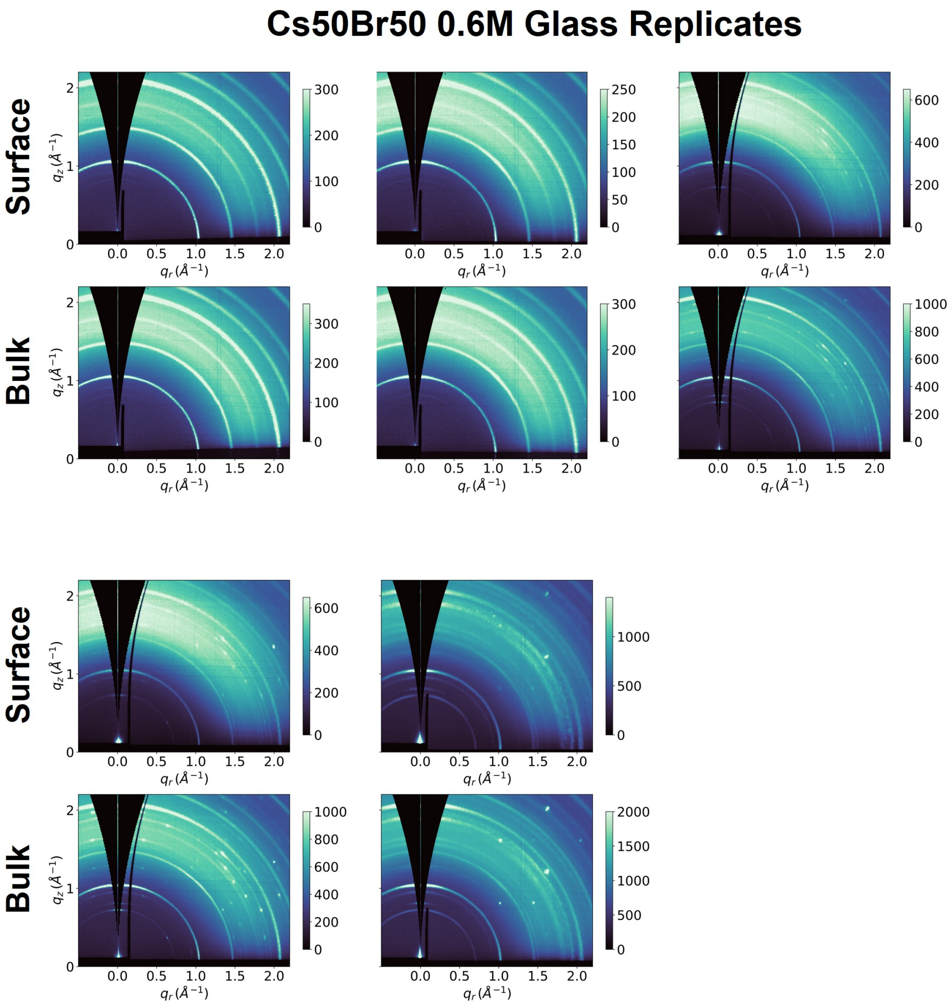


**Fig. S7** Glass 0.6 M Reproducibility Check - Cs50Br50. Replicates across samples and batches show same secondary phases present, with only slight variations in intensity or orientation.

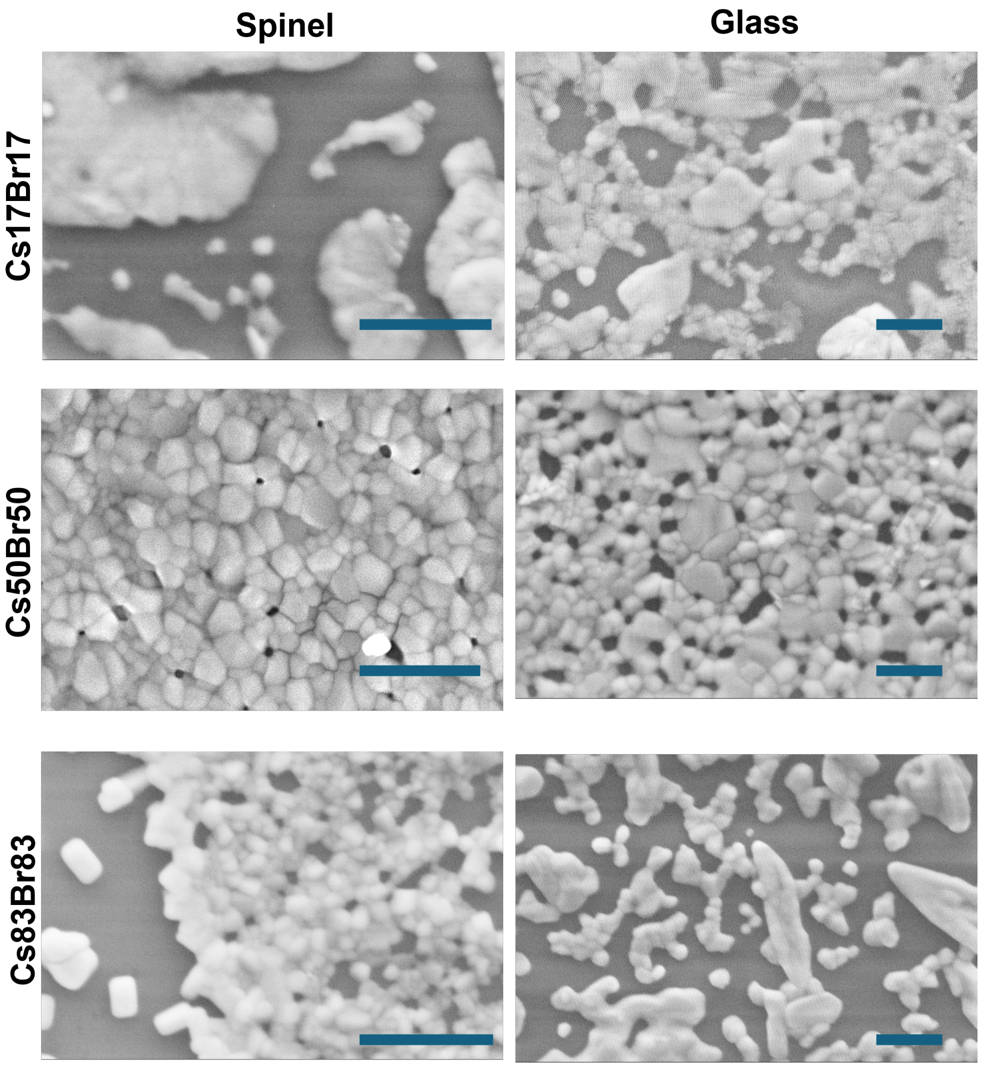


**Fig. S8** Full SEM Images from Figure 2. Scale bar = 1 *um*.

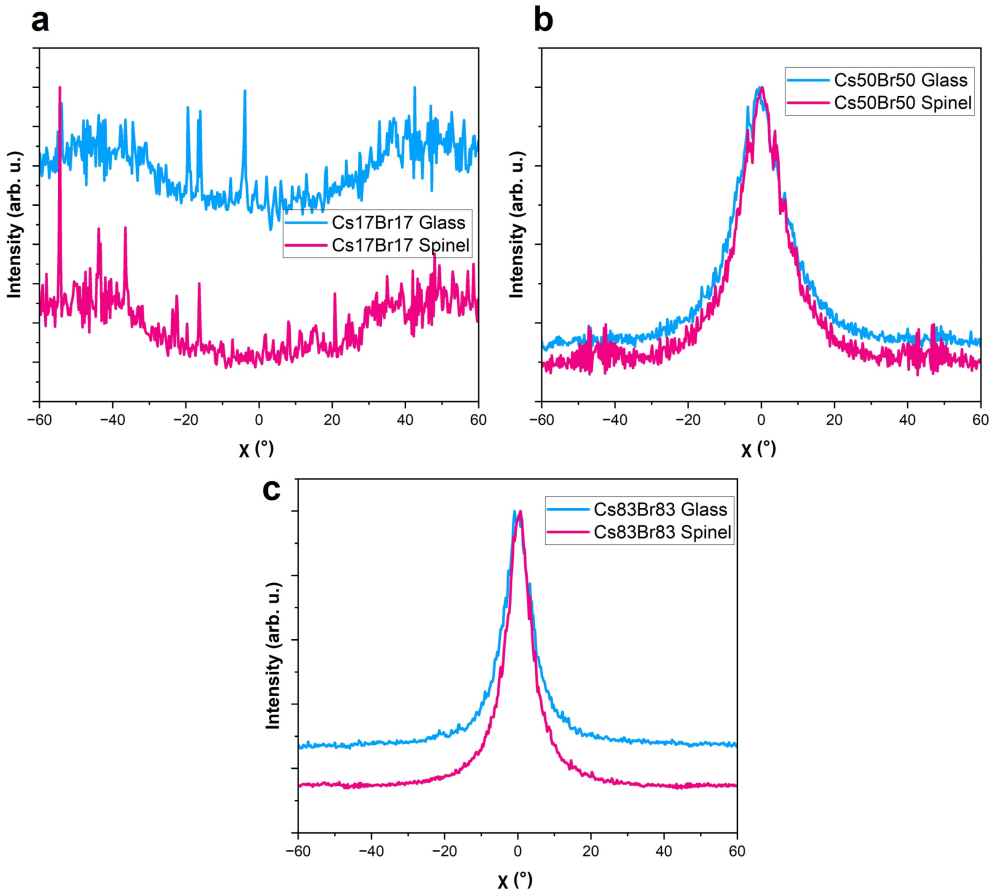


**Fig. S9** Azimuthal integrations of the (110) from 2D GIWAXS patterns of films made from 0.6 M precursor solutions on glass (pink) and $MgAl_2O_4$ (spinel, blue). $q_r$ is kept constant to integrate around feature but $q_r$ was changed for each composition to ensure correct peak was selected due to changes in lattice parameter with composition: a) Cs17Br17: $q_r = 1Å^{-1}$, b) Cs50Br50: $q_r = 1.05Å^{-1}$, and c) Cs83Br83: $q_r = 1.1Å^{-1}$ all with dq = 0.05°. Due to the configuration of grazing incidence measurements, the reconstruction of the Ewald sphere creates a missing wedge near $q_r = 0$ wherein there is no data available.[3, 4]

# 2 Supplemental Note 1: GIXRD Methods

GIXRD with varying $\psi$ is used to evaluate the residual strain in polycrystalline thin films by varying sample tilt. The highest order diffraction peak giving appreciable signal with contributions from multiple planes (i.e. (312)) should be used to most easily detect shifts due to strain. After collecting measurements with varying $\psi$, peak positions are converted to d-spacing using Bragg's law. Plotting peak positions as a function of $sin^2\psi$ and taking the slope allows for quantification of in-plane strain. The

method used is as follows, based upon works from McAndrews et. al, and Luo, et. al, [5–7].

Strain in the z-direction (planes parallel to the substrate) where $d_{hkl}$ is the measured lattice spacing and $d_0$ is the unstrained lattice spacing (assumed to be the measured lattice spacing at $\psi = 0$):

$$\epsilon_{zz} = \frac{\mathrm{d_{hkl}\text{-}d_0}}{\mathrm{d_0}} \tag{1}$$

The principal equation of the $sin^2\psi$ technique is as follows where $\nu$ is the Poisson's ratio, E is elastic modulus, $\phi$ is the in-plane stress with 2 components ($\sigma_1$ and $\sigma_2$ ), $\sigma_3$ is the out of plane stress $\psi$ is the sample tilt (0° = flat - X-ray is perpendicular, 90° - X-ray is parallel):

$$\frac{\mathrm{d_{hkl}\text{-}d_0}}{\mathrm{d_0}} = \epsilon_\psi = (\frac{1+\nu}{\mathrm{E}}) \cdot (\sigma_1 \sin^2\phi + \sigma_2 \cos^2\phi) \cdot \sin^2\psi + \frac{1}{\mathrm{E}}(\sigma_3 - \nu(\sigma_1 + \sigma_2)) \tag{2}$$

To simplify calculations we rely on two assumptions based on unconstrained expansion and contraction normal to the substrate: 1) Stress is equi-biaxial or independent of orientation ($\sigma_1 = \sigma_2$) and 2) the film is mechanically isotropic ($\sigma_3 = 0$).

These assumptions allow for the following simplification:

$$\epsilon_\psi = (\frac{1+\nu}{\mathrm{E}}) \cdot \sigma \cdot \sin^2\psi - (\frac{2\,\sigma\nu}{\mathrm{E}}) \tag{3}$$

Using the conversion between lattice spacing and strain $d_\psi = d_0(1 + \epsilon_\psi)$ the equation can then be put in terms of lattice spacing and stress:

$$\mathrm{d}_\psi = \mathrm{d_0}(1 + \epsilon_\psi) \tag{4}$$

Simplify:

$$\mathrm{d}_\psi = \mathrm{d_0} + \mathrm{d_0}\frac{1+\nu}{\mathrm{E}}\sigma \cdot \sin^2\psi - \mathrm{d_0} \cdot (\frac{2\,\sigma\nu}{\mathrm{E}}) \tag{5}$$

Put in terms of a linear equation Y = AX + B:

$$\mathrm{d}_\psi = \mathrm{A} \cdot \sin^2\psi + \mathrm{B}\ \text{where}\ \mathrm{A} = [\frac{1+\nu}{\mathrm{E}} \cdot \sigma \cdot \mathrm{d_0}]\ \text{and}\ \mathrm{B} = [\mathrm{d_0} \cdot (1 - (\frac{2\,\nu\sigma}{\mathrm{E}})] \tag{6}$$

By plotting $d_\psi$ vs. $sin^2\psi$, a linear regression will yield the *slope (m)*, which can be input to the following equation to calculate absolute strain values *(with the assumption* $d_0 = d_{\psi=0}$*)*:

$$\sigma = (\frac{\mathrm{E}}{1+\nu})(\frac{\mathrm{m}}{\mathrm{d_0}}) \tag{7}$$

For works where relative strain is of more importance than absolute values, the slope $m$ is commonly used for comparison.[8–11] In this work, we fitted slopes using a linear regression model for each composition on the spinel and glass.

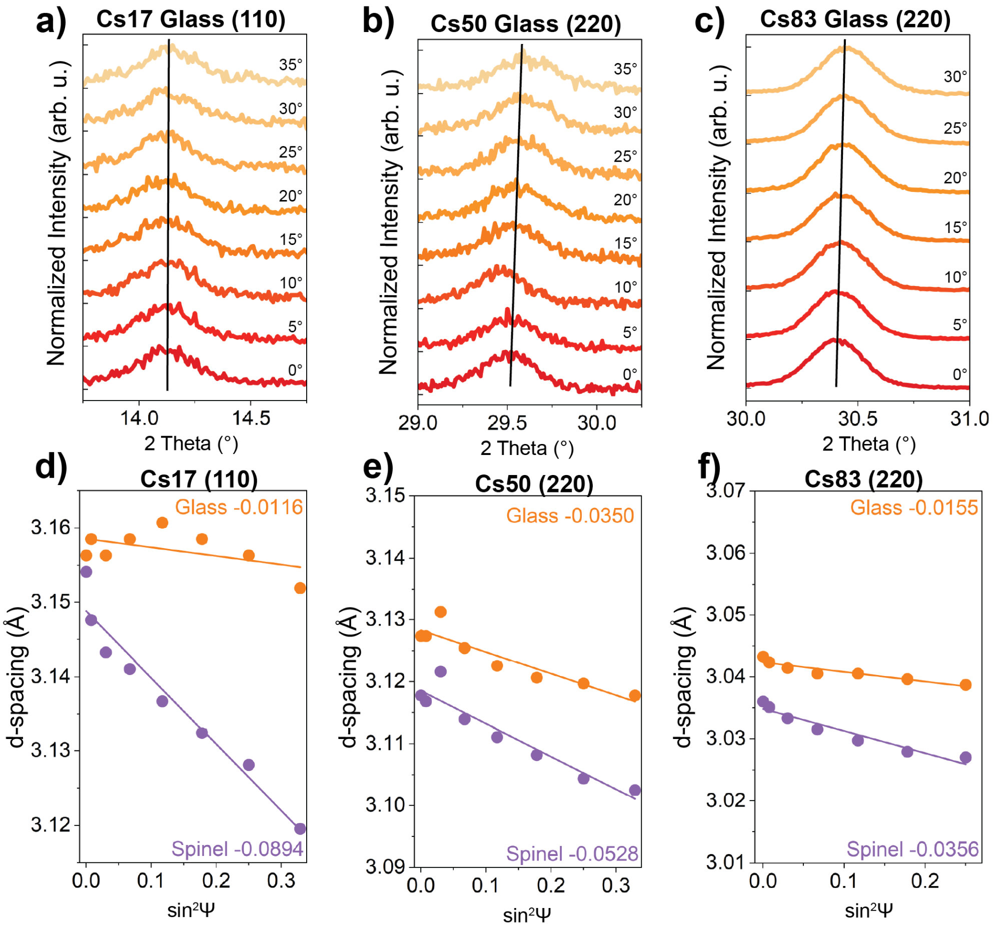


**Fig. S10** $sin^2\psi$ Measurements on Glass. Peak position as a function of $\psi$ for a) Cs17Br17, b) Cs50Br50, and c) Cs83Br83. d-f) Fitted peak positions are plotted as a function of $sin^2\psi$ and fit with linear regression for glass and spinel. All slopes are negative, indicating compressive strain with significantly higher strain in films on $MgAl_2O_4$. Strain in glass films could be due to thermal annealing, but is notably less than that on lattice matched substrates.

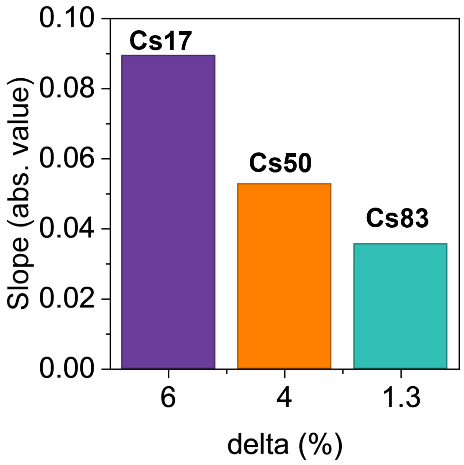


**Fig. S11** Slope versus lattice mismatch ($\delta$). Greater $\delta$ corresponds with greater slope (compressive strain) supporting mismatch-dependent lattice coupling-induced strain.

**Table S3** Film Thickness as a Function of Precursor Solution Concentration Measured By Profilometry

| $Cs_{0.50}Br_{0.50}$ **Precursor Solution Molarity** | **Film Thickness** | |
|---|---|---|
| | Spinel ($MgAl_2O_4$) | Glass |
| 0.8 M | 130 nm | 110 nm |
| 0.6 M | 78 nm | 83 nm |
| 0.4 M | 46 nm | 46 nm |

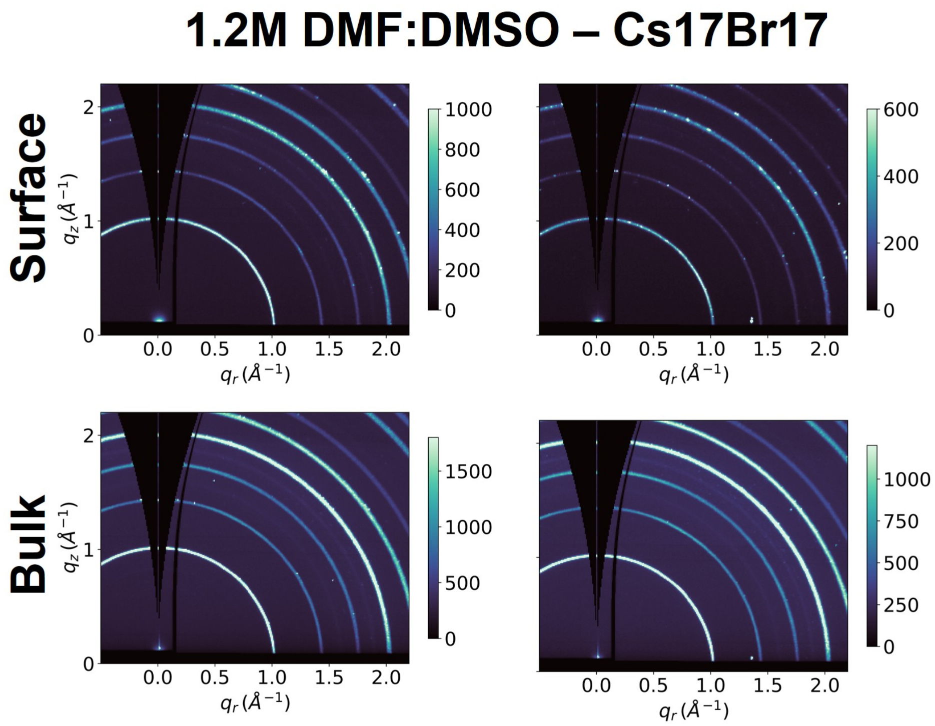


**Fig. S12** Spinel Thickness and Reproducibility Check - Cs17Br17. There is no change in the effect of the spinel $MgAl_2O_4$ with film thickness, through the thickness used in a full solar cell device.

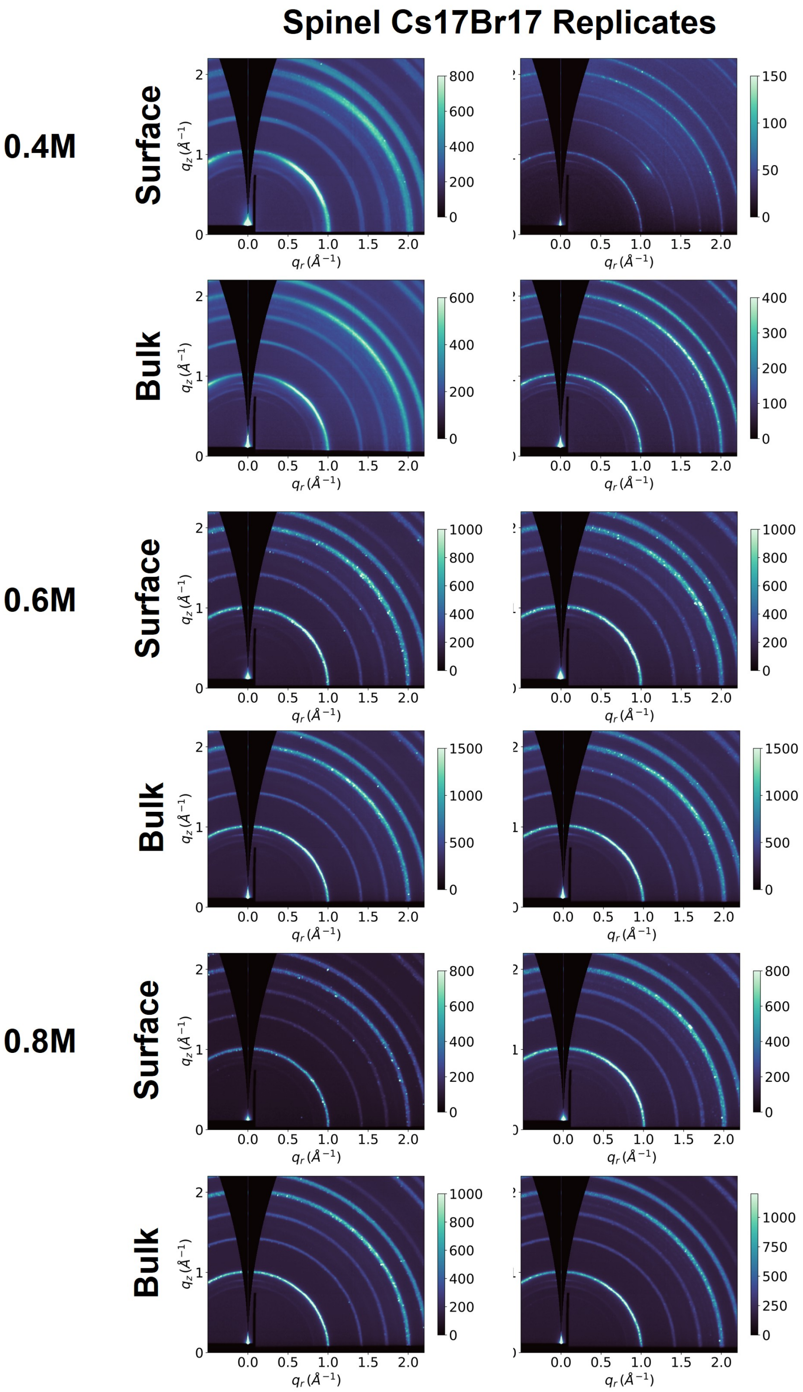


**Fig. S13** Spinel Thickness and Reproducibility Check - Cs17Br17. There is no change in the effect of the spinel $MgAl_2O_4$ with film thickness or across replicates.

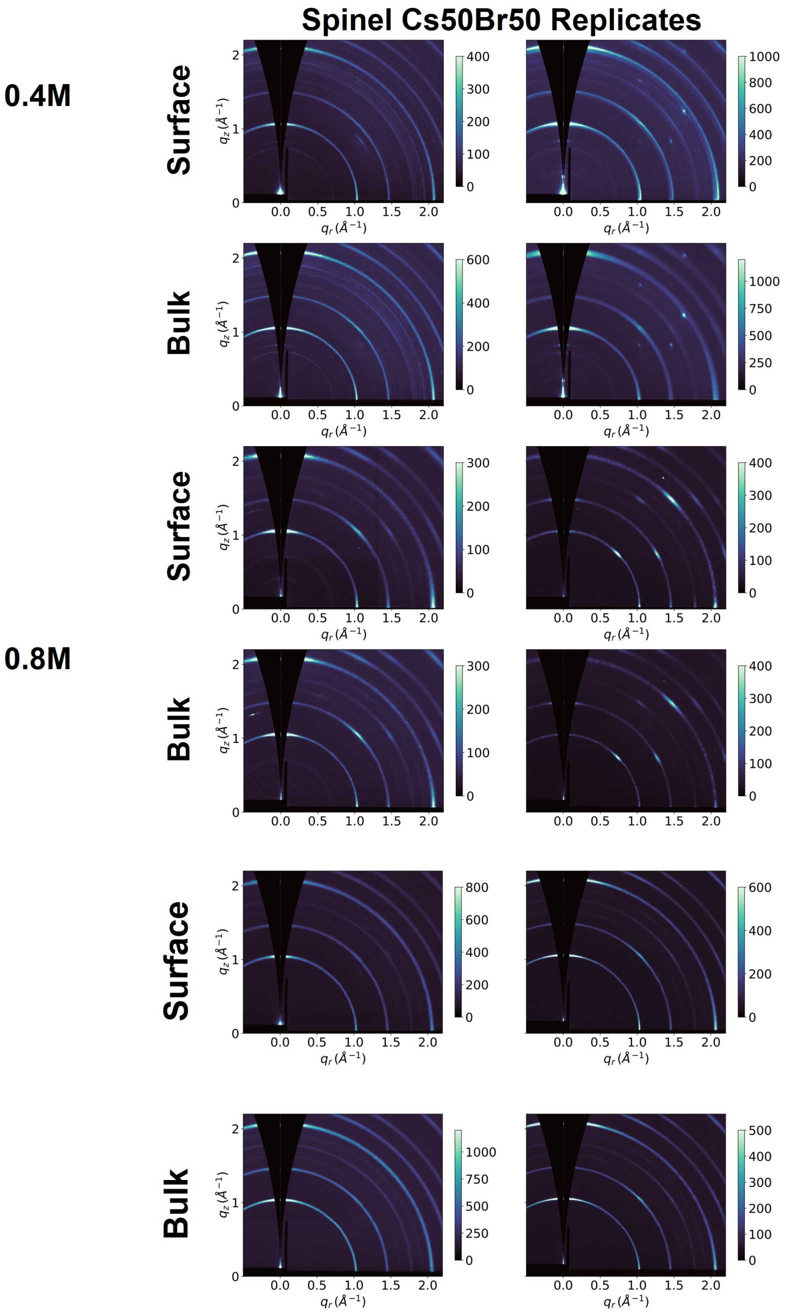


**Fig. S14** Spinel Thickness and Reproducibility Check - Cs50Br50. There is no change in the effect of the spinel $MgAl_2O_4$ with film thickness or across replicates.

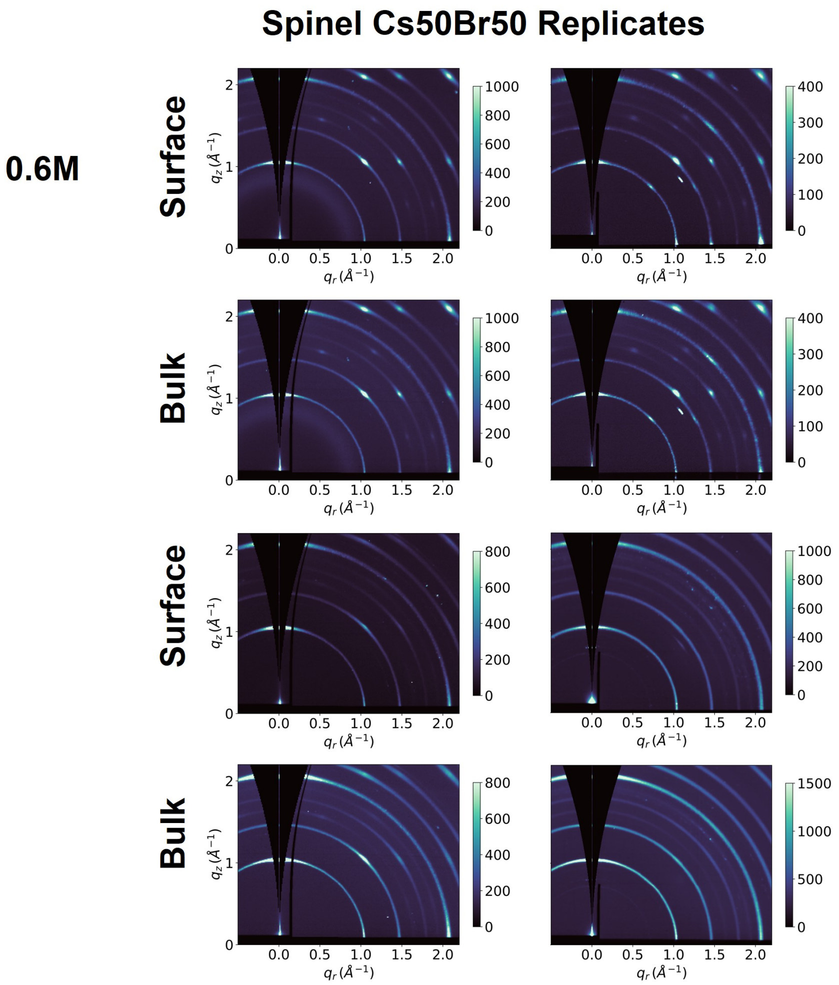


**Fig. S15** Spinel Thickness and Reproducibility Check - Cs50Br50. There is no change in the effect of the spinel $MgAl_2O_4$ with film thickness or across replicates.

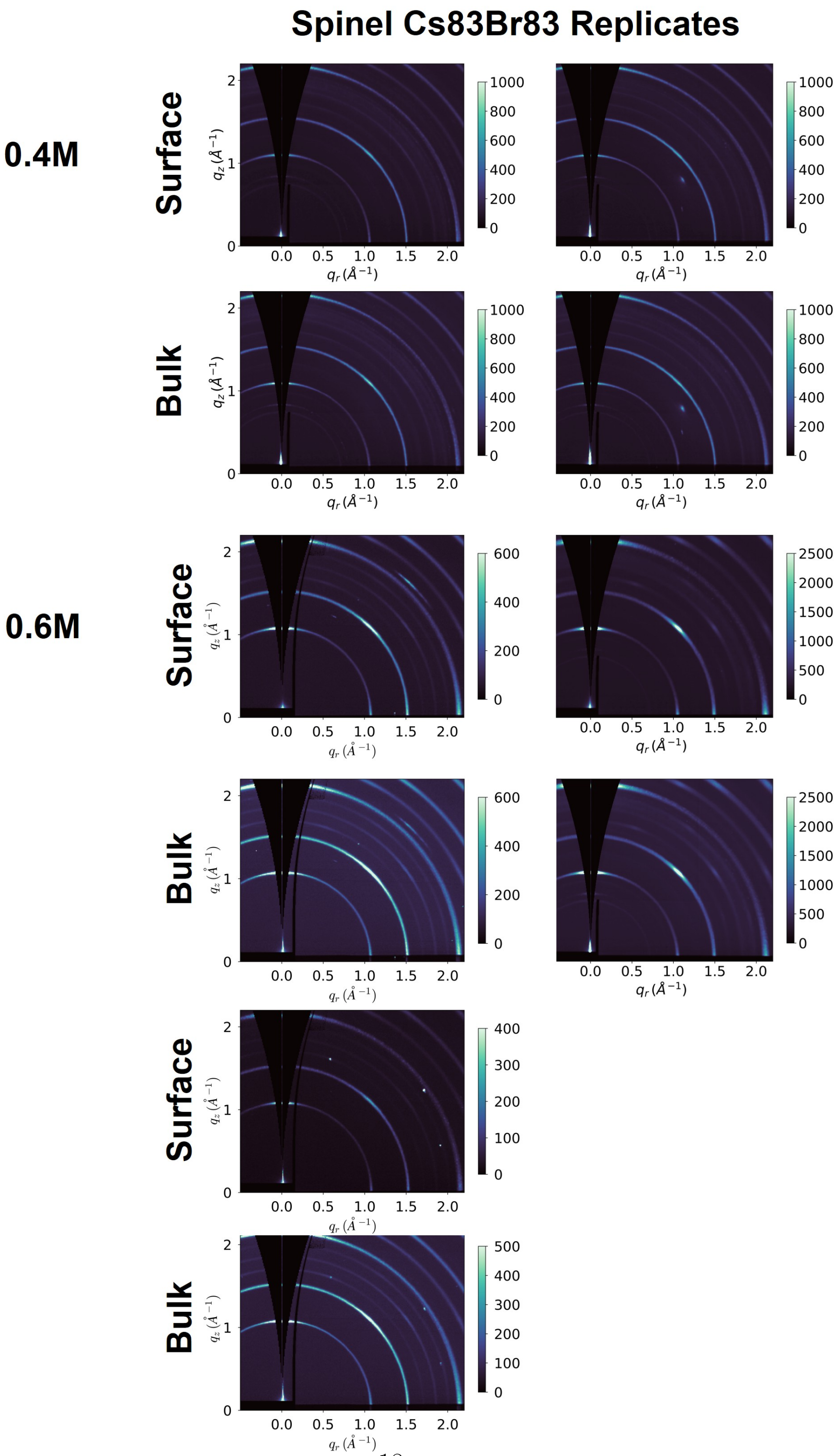




**Fig. S16** Spinel Thickness and Reproducibility Check - Cs83Br83. There is no change in the effect of the spinel $MgAl_2O_4$ with film thickness or across replicates.

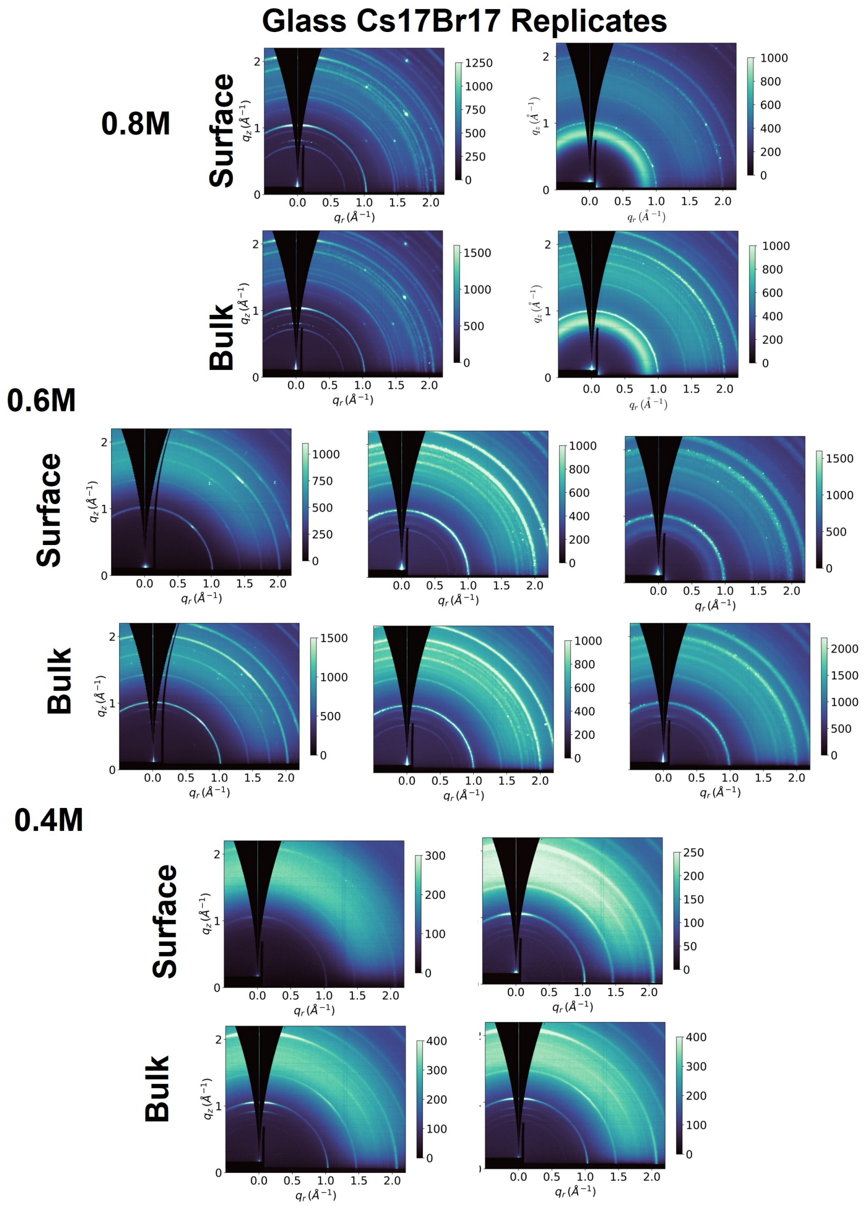


**Fig. S17** Glass Thickness and Reproducibility Check - Cs17Br17. Secondary phases are present across all thicknesses and replicates, with only small changes in relative intensities of the different phases.

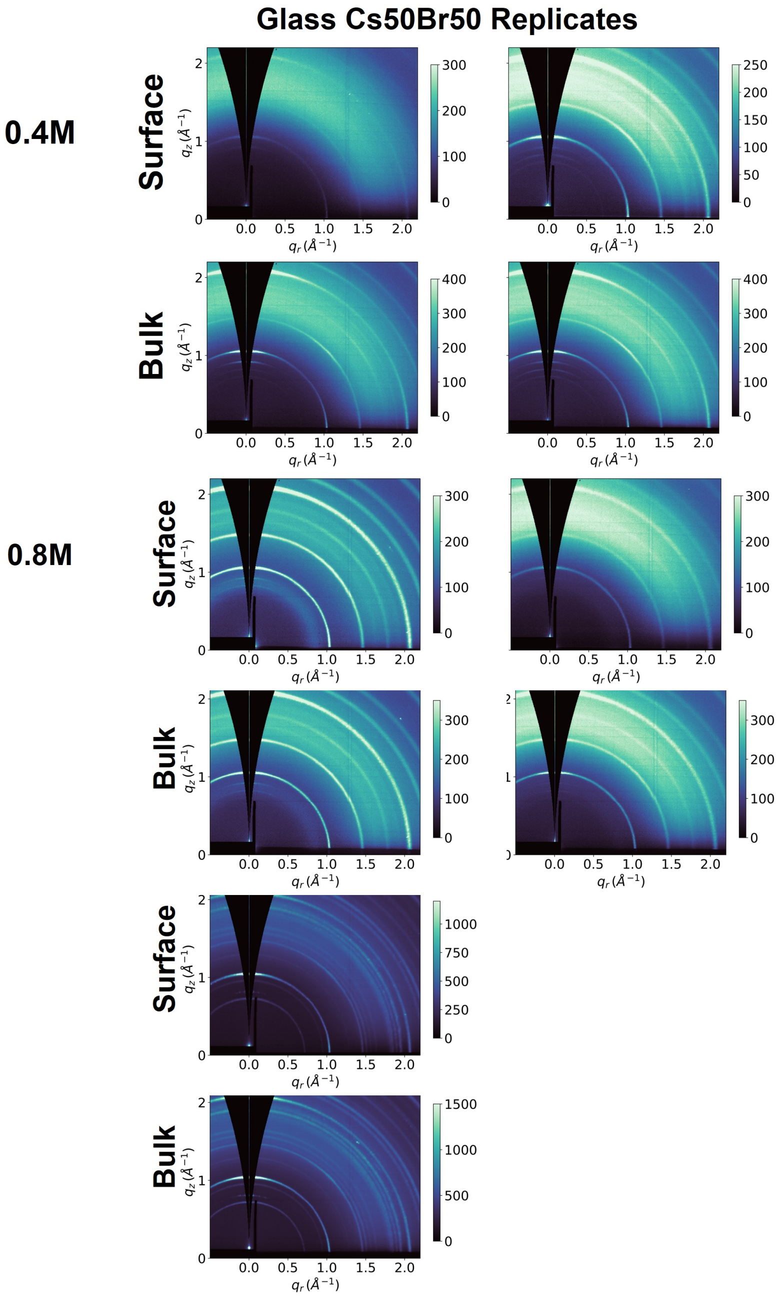


**Fig. S18** Glass Thickness and Reproducibility Check - Cs50Br50. Secondary phases are present across all thicknesses and replicates, with only small changes in relative intensities of the different phases.

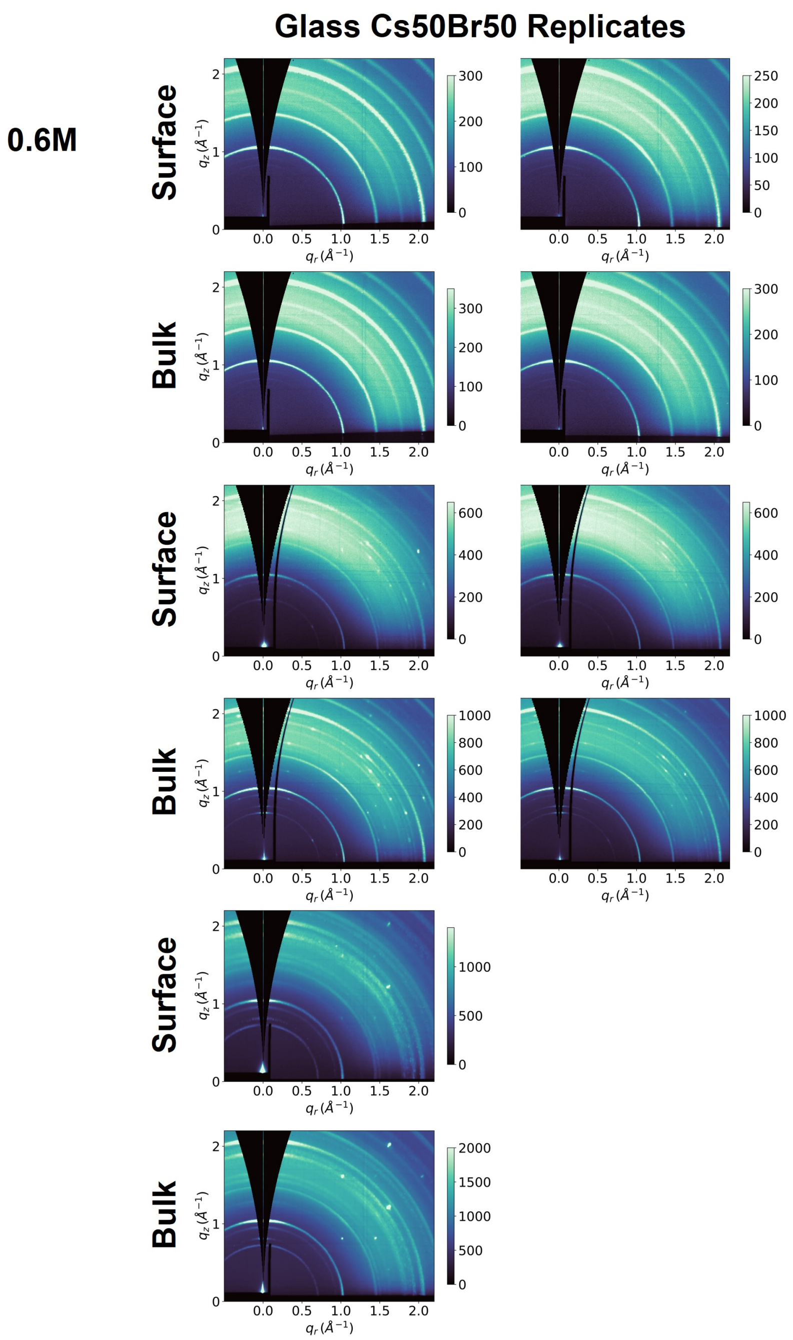


**Fig. S19** Glass Thickness and Reproducibility Check - Cs50Br50. Secondary phases are present across all thicknesses and replicates, with only small changes in relative intensities of the different phases.

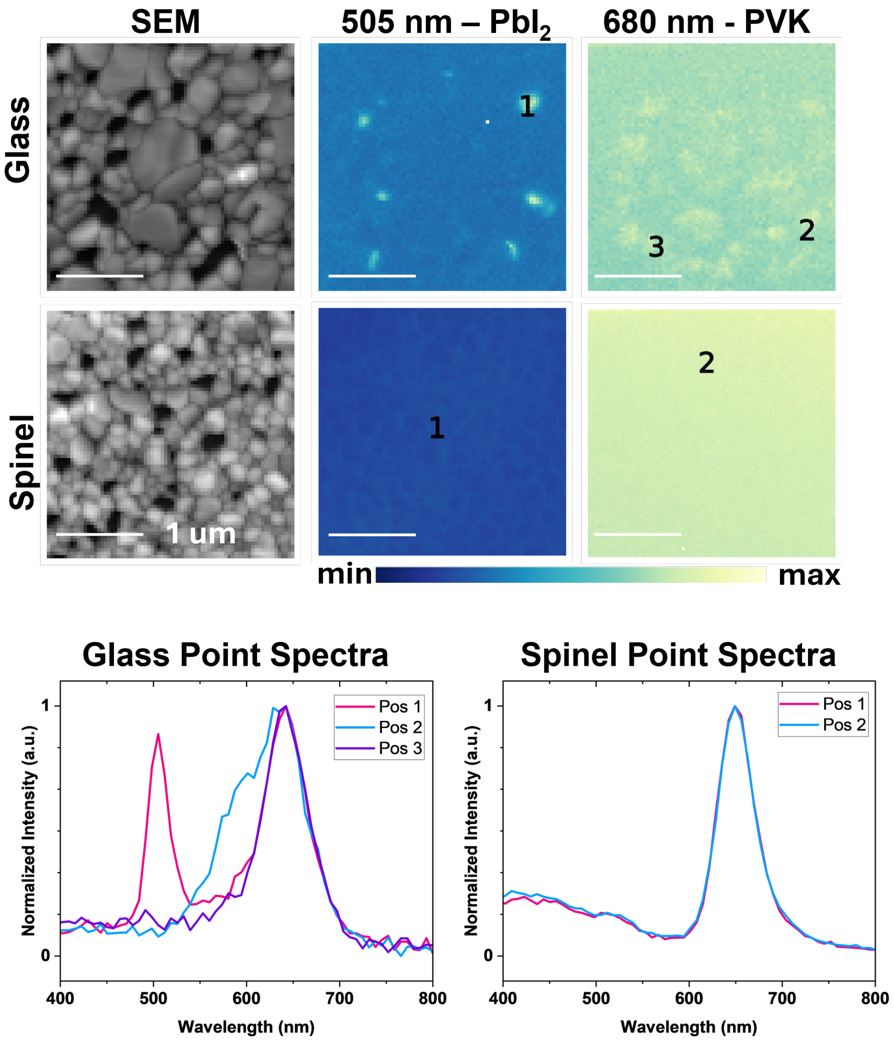


**Fig. S20** CL-SEM of Cs50Br50 0.6 M films shown in Figure 3. $PbI_2$ overlays in Figure 3 are extracted from $PbI_2$ maps here. Scale bar is 1 um. Point spectra show representative CL spectra from the marked points.

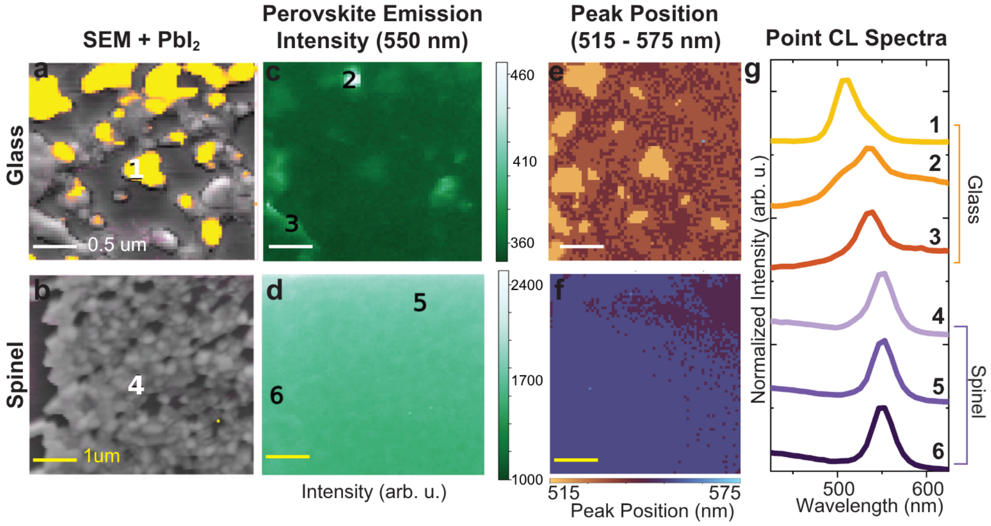


**Fig. S21** CL-SEM of Cs83Br83 0.6 M films on glass and $MgAl_2O_4$ (spinel). a,b) SEM images with extracted $PbI_2$ overlays that show LHP films on $MgAl_2O_4$ have less $PbI_2$. c,d) CL intensity maps of characteristic perovskite emission (550 nm) intensity (note difference in scale bars showing higher intensity for LHP on spinel oxide). e,f) Maps of perovskite peak emission position between 515-575 nm. g) Normalized point CL spectra at representative points - glass (orange) and $MgAl_2O_4$ (spinel, purple).

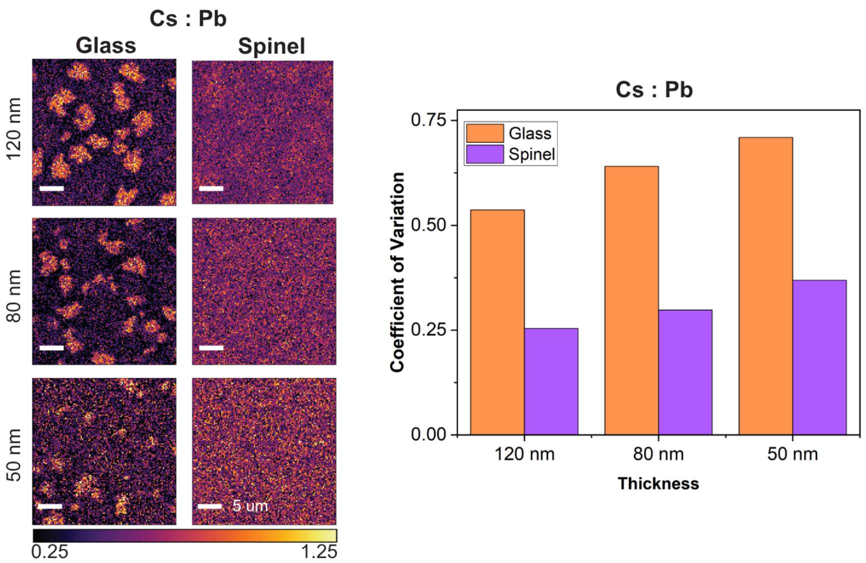


**Fig. S22** XRF Maps and Coefficient of Variation of Cs:Pb Elemental Maps as a function of thickness.

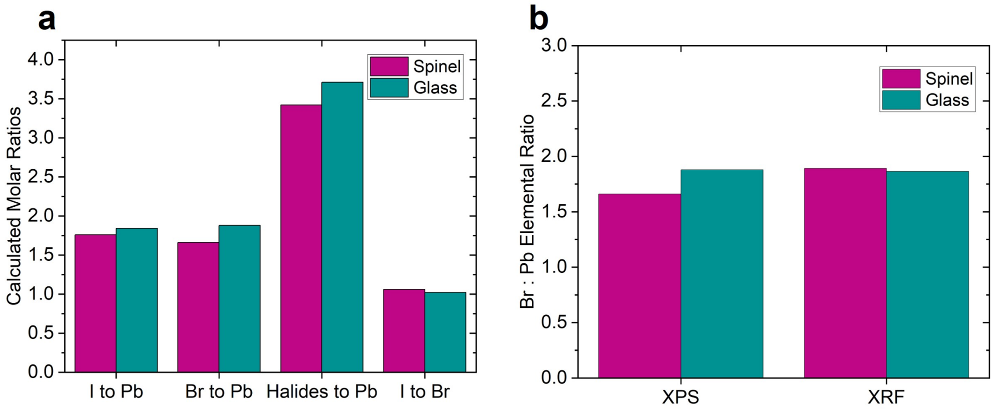


**Fig. S23** Comparing Surface and Bulk Elemental Composition a) Elemental Ratios from XPS for $MgAl_2O_4$ (spinel, pink) and glass (teal). b) Comparing Br:Pb ratios for XPS and XRF. Br:Pb is the only ratio that has no closely overlapping fluorescence edges, so XRF ratios can be directly compared with XPS. Overlapping Cs and I Ledges in XRF make ratios containing those elements difficult to compare across techniques.

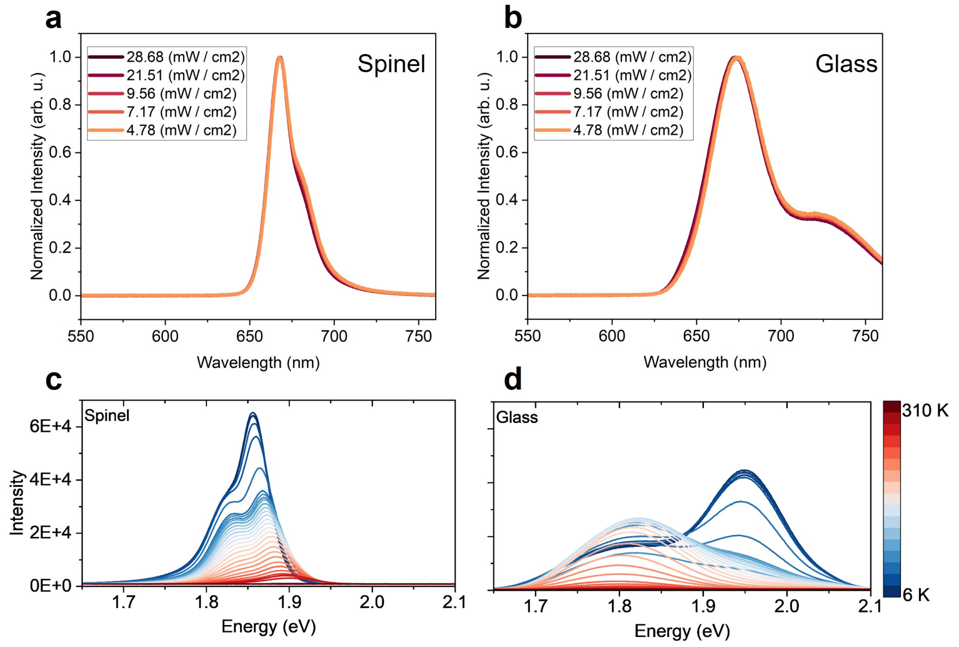


**Fig. S24** Power Dependence of Cryogenic PL for 0.6 M Cs50Br50 on a) $MgAl_2O_4$ (spinel) and b) glass. Unnormalized temperature dependent PL for films on c) $MgAl_2O_4$ (spinel) and d) glass. Peak positions in Figure 5g,h were found by fitting 2 Gaussians.

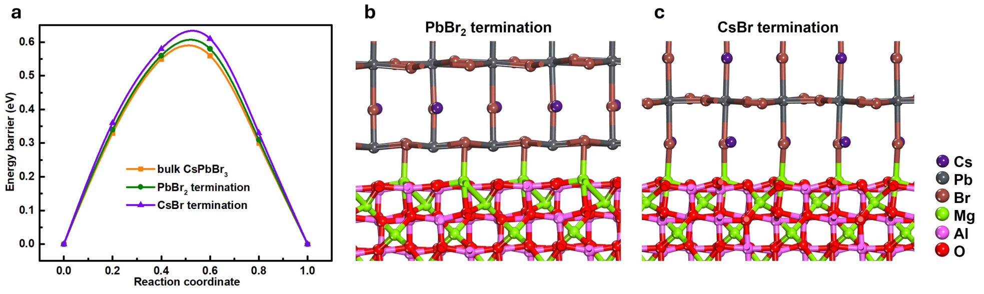


**Fig. S25** a) Bromide diffusion barriers from NEB calculations in the b) $PbBr_2$ termination and c) CsBr termination. The vacancy formation energies are 2.82 eV (bulk $CsPbBr_3$), 2.86 eV ($PbBr_2$ termination), and 2.91 eV (CsBr termination).

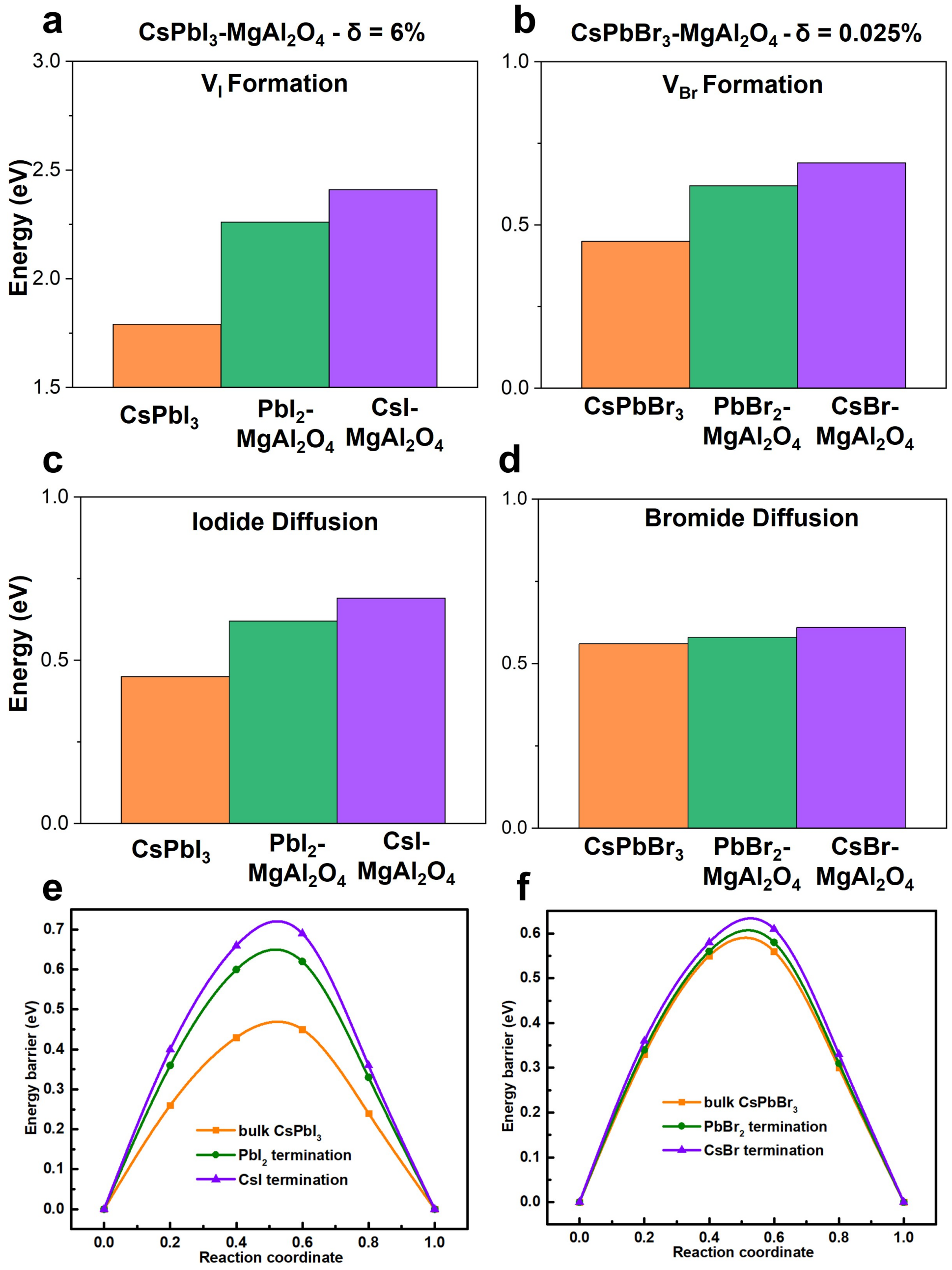


**Fig. S26** Comparison of DFT calculations with varying degrees of lattice mismatch. Energy for halide vacancy formation for a) $CsPbI_3$ and b) $CsPbBr_3$ that shows both lattice matched systems require more energy to form halide vacancies than the pristine perovskite. c-f) Energetic barriers for halide diffusion show that the spinel oxide interface has a greater effect with an increased $\delta$, indicating the key role of mismatch-dependent compressive strain in preventing ion migration.

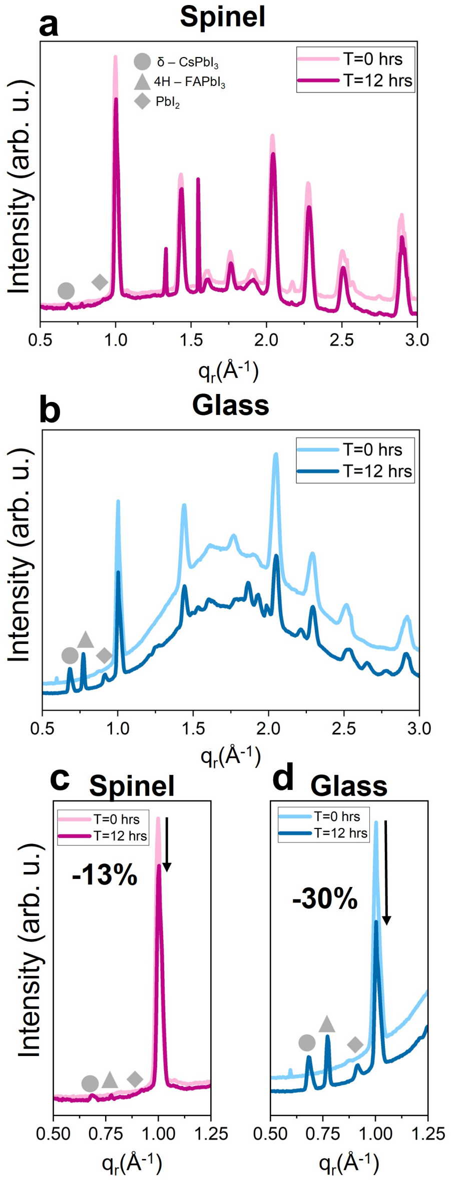


**Fig. S27** GIWAXS Circular Averages of Initial and Final In-situ Humidity Cs50Br50 films from 0.6 M precursor solutions on a) $MgAl_2O_4$ (spinel) and b) glass. c,d) Zoom in on low angle peaks to show suppression of secondary phase formation on $MgAl_2O_4$ (spinel). Perovskite peak intensity decreases by only 13% on $MgAl_2O_4$ (spinel) as compared to 30% on glass. The lighter shade is T = 0 and the darker shade is T = 12 hours. Sharp peaks around $q_r = 1.5\,\text{Å}^{-1}$ on the spinel are the substrate itself.

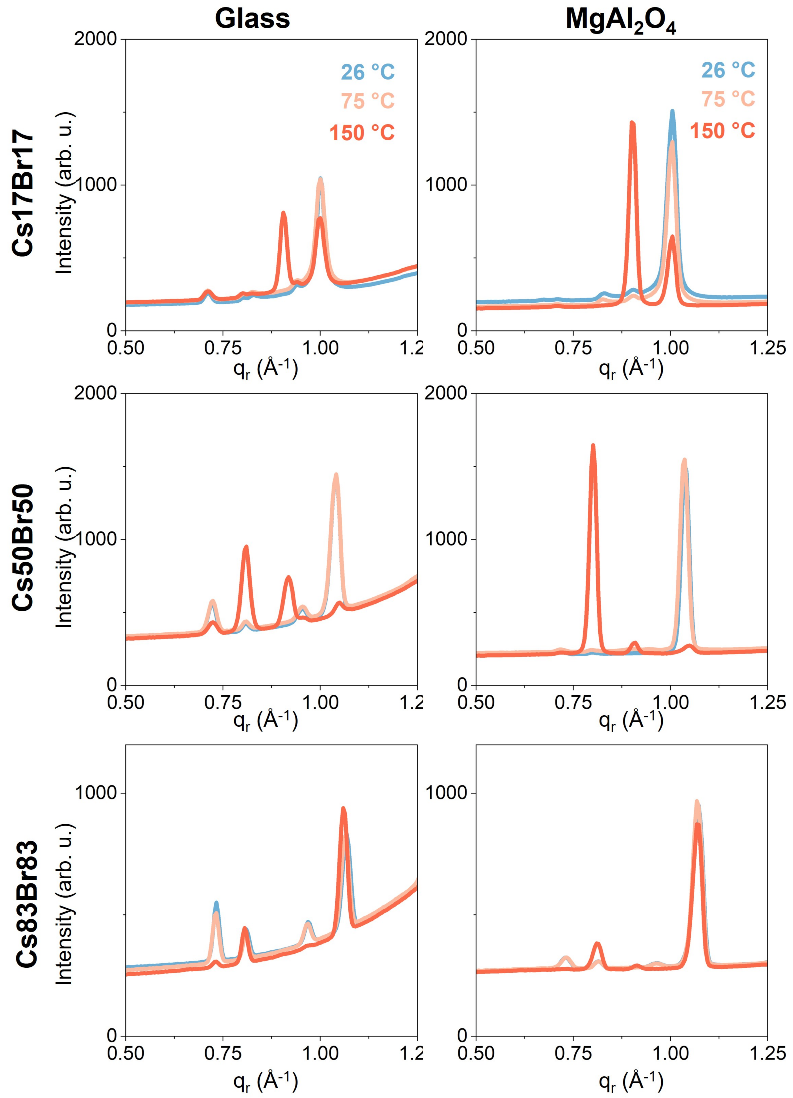


**Fig. S28** In-situ temperature GIWAXS of LHPs with varying degrees of lattice mismatch for 80 nm films on glass and spinel oxides. Films were first measured at room temperature, held at 75 °C for 45 min, then held at 150 °C for 45 minutes. Degradation at high temperatures is shown by the loss of the perovskite peak around $q_r = 1\,\text{Å}^{-1}$ and formation of undesirable secondary phases below $q_r = 1\,\text{Å}^{-1}$. All films degrade above 75 °C, as is expected due to the likely loss of organic and halides from mixed-cation mixed-halide films above 100 °C.[12, 13] Instead, thermal stability of the perovskite phase seems to be primarily dictated by chemistry. Increasing Cs concentration improves thermal stability on either substrate, supporting a mechanism of thermal stability driven by loss of organic and halides.

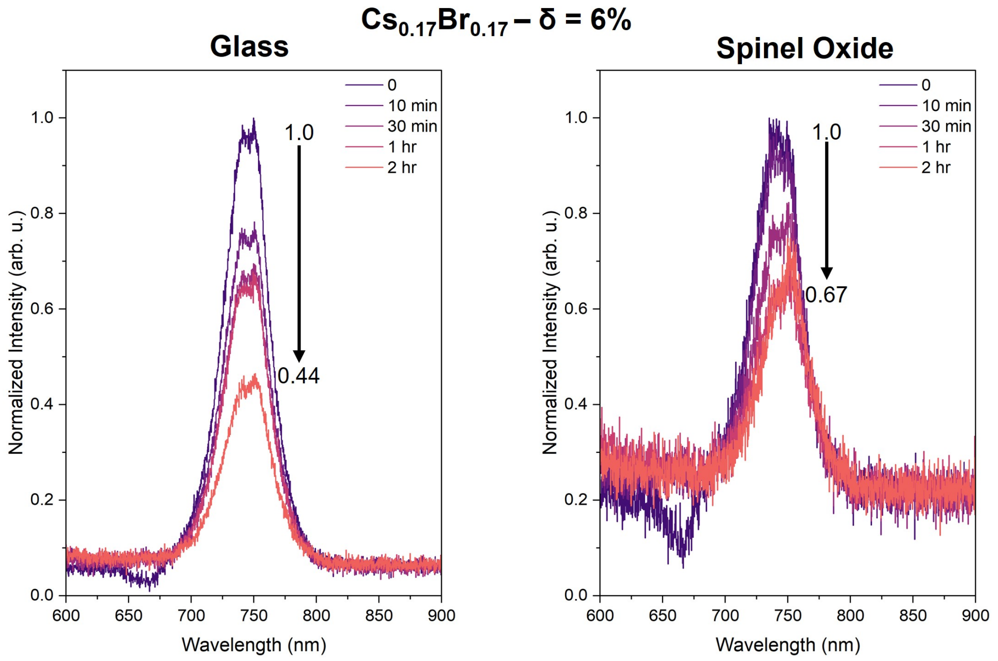


**Fig. S29** Stability of LHP thin films under 0.5 Sun illumination measured by photoluminescence. The magnitude of PL quenching is more significant for glass (-66%) than spinel oxide (-33%), indicated improved stability under illumination after modifying the substrate.

# References


[1] Brik, M. G., Suchocki, A. & Kamińska, A. Lattice Parameters and Stability of the Spinel Compounds in Relation to the Ionic Radii and Electronegativities of Constituting Chemical Elements. *Inorganic Chemistry* **53**, 5088–5099 (2014). URL https://doi.org/10.1021/ic500200a. Publisher: American Chemical Society.

[2] An, Y. *et al.* Identifying high-performance and durable methylammonium-free lead halide perovskites via high-throughput synthesis and characterization. *Energy & Environmental Science* **14**, 6638–6654 (2021). URL https://pubs.rsc.org/en/content/articlehtml/2021/ee/d1ee02691g. Publisher: The Royal Society of Chemistry.

[3] Steele, J. A. *et al.* How to GIWAXS: Grazing Incidence Wide Angle X-Ray Scattering Applied to Metal Halide Perovskite Thin Films. *Advanced Energy Materials* **13**, 2300760 (2023). URL https://onlinelibrary.wiley.com/doi/abs/10.1002/aenm.202300760.

[4] Hidalgo, J. *et al.* Solvent and A-Site Cation Control Preferred Crystallographic Orientation in Bromine-Based Perovskite Thin Films. *Chemistry of Materials* **35**, 4181–4191 (2023). URL https://doi.org/10.1021/acs.chemmater.3c00075. Publisher: American Chemical Society.

[5] McAndrews, G. R., Guo, B., Morales, D. A., Amassian, A. & McGehee, M. D. How the dynamics of attachment to the substrate influence stress in metal halide perovskites. *APL Energy* **1**, 036110 (2023). URL https://doi.org/10.1063/5.0177697.

[6] Luo, Q. A modified X-ray diffraction method to measure residual normal and shear stresses of machined surfaces. *The International Journal of Advanced Manufacturing Technology* **119**, 3595–3606 (2022). URL https://doi.org/10.1007/s00170-021-08645-4.

[7] Luo, Q. & Jones, A. H. High-precision determination of residual stress of polycrystalline coatings using optimised XRD-sin2 technique. *Surface and Coatings Technology* **205**, 1403–1408 (2010). URL https://www.sciencedirect.com/science/article/pii/S0257897210006304.

[8] Lee, D.-K. *et al.* Strain Engineering: Reduction of Microstrain at the Perovskite Surface via Alkali Metal Chloride Treatment Enhances Stability. *ACS Energy Letters* **10**, 1039–1049 (2025). URL https://doi.org/10.1021/acsenergylett.4c03334. Publisher: American Chemical Society.

[9] Chen, Y. *et al.* Interface-oriented bridges toward efficient carbon-based perovskite solar cells. *Nanoscale* (2025). URL https://pubs.rsc.org/en/content/articlelanding/2025/nr/d4nr04719b.

[10] Xue, D.-J. *et al.* Regulating strain in perovskite thin films through charge-transport layers. *Nature Communications* **11**, 1514 (2020). URL https://www.nature.com/articles/s41467-020-15338-1. Publisher: Nature Publishing Group.

[11] Zhu, C. *et al.* Strain engineering in perovskite solar cells and its impacts on carrier dynamics. *Nature Communications* **10**, 815 (2019). URL https://www.nature.com/articles/s41467-019-08507-4.

[12] LaFollette, D. K. *et al.* Bromine Incorporation Affects Phase Transformations and Thermal Stability of Lead Halide Perovskites. *Journal of the American Chemical Society* **146**, 18576–18585 (2024). URL https://doi.org/10.1021/jacs.4c04508. Publisher: American Chemical Society.

[13] Long, M. *et al.* Abnormal Synergetic Effect of Organic and Halide Ions on the Stability and Optoelectronic Properties of a Mixed Perovskite via In Situ Characterizations. *Advanced Materials* **30**, 1801562 (2018). URL https://onlinelibrary.wiley.com/doi/abs/10.1002/adma.201801562.
